# Cyp1b1 Regulates Ocular Fissure Closure Through a Retinoic Acid–Independent Pathway

**DOI:** 10.1167/iovs.16-20235

**Published:** 2017-02

**Authors:** Antionette L. Williams, Jessica Eason, Bahaar Chawla, Brenda L. Bohnsack

**Affiliations:** Department of Ophthalmology and Visual Sciences, Kellogg Eye Center, University of Michigan, Ann Arbor, Michigan, United States

**Keywords:** congenital glaucoma, neural crest, CYP1B1, retinoic acid, coloboma

## Abstract

**Purpose:**

Mutations in the *CYP1B1* gene are the most commonly identified genetic causes of primary infantile-onset glaucoma. Despite this disease association, the role of *CYP1B1* in eye development and its in vivo substrate remain unknown. In the present study, we used zebrafish to elucidate the mechanism by which *cyp1b1* regulates eye development.

**Methods:**

Zebrafish eye and neural crest development were analyzed using live imaging of transgenic zebrafish embryos, in situ hybridization, immunostaining, TUNEL assay, and methylacrylate sections. *Cyp1b1* and retinoic acid (RA) levels were genetically (morpholino oligonucleotide antisense and mRNA) and pharmacologically manipulated to examine gene function.

**Results:**

Using zebrafish, we observed that *cyp1b1* was expressed in a specific spatiotemporal pattern in the ocular fissures of the developing zebrafish retina and regulated fissure patency. Decreased Cyp1b1 resulted in the premature breakdown of laminin in the ventral fissure and altered subsequent neural crest migration into the anterior segment. In contrast, *cyp1b1* overexpression inhibited cell survival in the ventral ocular fissure and prevented fissure closure via an RA-independent pathway. *Cyp1b1* overexpression also inhibited the ocular expression of *vsx2*, *pax6a*, and *pax6b* and increased the extraocular expression of *shha*. Importantly, embryos injected with human wild-type but not mutant *CYP1B1* mRNA also showed colobomas, demonstrating the evolutionary and functional conservation of gene function between species.

**Conclusions:**

*Cyp1b1* regulation of ocular fissure closure indirectly affects neural crest migration and development through an RA-independent pathway. These studies provide insight into the role of Cyp1b1 in eye development and further elucidate the pathogenesis of primary infantile-onset glaucoma.

In primary infantile-onset glaucoma (Online Mendelian Inheritance in Man [OMIM] 231300), elevated intraocular pressures resulting from the malformation of the trabecular meshwork lead to irreversible damage to the optic nerve, cornea, and sclera. Children with primary infantile-onset glaucoma often require multiple eye surgeries; however, eye pressures can be difficult to control, and many affected individuals are blind.^1,2^ Autosomal recessive mutations in the *CYP1B1* gene are the most commonly identified genetic causes of primary infantile-onset glaucoma.^3^ Despite its association with human disease, the role of *CYP1B1* in eye development remains unknown. *Cyp1b1* knockout mice showed angle abnormalities involving Schlemm's canal, trabecular meshwork, cornea, and iris; yet these defects did not lead to elevations in intraocular pressure.^4^ Additional animal studies are required to understand the function of *CYP1B1* during embryogenesis.

The *CYP1B1* gene encodes a cytochrome p450 enzyme that regulates the in vitro synthesis of the essential morphogen retinoic acid (RA) through the conversion of retinol (vitamin A) into retinaldehyde and subsequently retinaldehyde into RA in a pathway independent of aldehyde dehydrogenases.^5^ Similar to other RA synthesis enzymes (e.g., *raldh2* [*aldh1a2*] and *raldh3* [*aldh1a3*]), *cyp1b1* is expressed in the developing dorsal and ventral retina. In contrast, RA degradation enzymes, including *cyp26a1*, *cyp26b1*, and *cyp26c1*, are localized to the cranial and caudal retina.^6–9^ Together, these enzymes create RA gradients in the periocular mesenchyme centered on the dorsal and ventral axis of the eye.

We previously demonstrated that in zebrafish, RA produced by the developing retina regulates the formation of neural crest–derived structures in the eye and craniofacial region.^10,11^ The neural crest is a transient population of stem cells that contribute to many structures in the anterior segment, including the corneal stroma and endothelium, sclera, iris stroma, ciliary body stroma, and trabecular meshwork.^12,13^ The genetic or toxic disruption of ocular neural crest migration, proliferation, survival, and differentiation is responsible for many congenital eye diseases, including primary infantile-onset glaucoma, Axenfeld-Rieger syndrome (OMIM 180500), and Peters anomaly (OMIM 604229).^14–16^ Thus, investigating the molecular regulation of neural crest development is important for understanding the basis of congenital eye diseases.

In the present study, we demonstrated that *cyp1b1* is expressed in the dorsal and ventral retina and retinal pigment epithelium, specifically in the ocular fissures, and maintains fissure patency, such that decreased expression caused premature closure, while overexpression resulted in colobomas. Although *cyp1b1* regulated local RA levels, the effect on fissure closure was independent of RA. *Cyp1b1* regulated the expression of *vsx2*, *pax6*, and *shh*, genes clinically associated with colobomas. Importantly, we demonstrated evolutionary and functional conservation between the human and zebrafish forms of *cyp1b1*. These studies better define the role of Cyp1b1 in eye development and, importantly, provide insight into the pathogenesis of primary infantile-onset glaucoma.

## Materials and Methods

### Animal Care and Animal Strains

Zebrafish (*Danio rerio*) were raised in a breeding colony under a 14-hour light/10-hour dark cycle as previously described.^10,11,17,18^ Embryos were maintained at 28.5°C and staged as previously described.^19^ The transgenic strains Tg(*sox10:EGFP*) and Tg(*foxd3:GFP*) were gifts from Thomas Schilling and Mary Halloran. The strains were crossed into the *casper* (*roy*^−/−^, *nacre*^−/−^) background as indicated.^20–22^ The Tg(*RARE:mCHERRY*) transgenic strain was generated using Gateway cloning techniques as previously described.^23^ Animal protocols were performed in accordance with the guidelines for the humane treatment of laboratory animals established by the University of Michigan Committee on the Use and Care of Animals (IACUC, protocol 10205) and the ARVO Statement for the Use of Animals in Ophthalmic and Vision Research.

### Morpholino Oligonucleotides and mRNA Injections

Translation blocking, 5-base pair mismatch, and standard control antisense morpholino oligonucleotides (MO; Gene Tools, LLC, Corvallis, OR, USA) directed against zebrafish Cyp1b1 (ATG and 5′ UTR targeted) and Raldh2 (ATG and 5′ UTR targeted) (Supplementary Table S1) were reconstituted in deionized water. Concentrations yielding consistent and reproducible phenotypes were determined for each MO. One-cell-stage embryos were injected with 1 to 2 nL MO at a concentration of 0.1 to 0.25 mM (2.1–2.4 ng/nL).

Two nonoverlapping translation blocking MO against Cyp1b1 were used to confirm the observed phenotype. The ATG-targeted MO against Cyp1b1 was previously verified for efficacy by confirming decreased protein expression, but this antibody is no longer available.^24^ No commercially available antibodies against human *CYP1B1* with predicted cross-reactivity with zebrafish *cyp1b1* showed consistent detection of the zebrafish protein by Western blot. The same ATG-targeted MO sequence and same range of concentrations as were used by Timme-Laragy et al.^24^ were used in these experiments. While the ATG-targeted Cyp1b1 MO is insensitive to the human mRNA coding sequence, there is overlap with the initial part of the zebrafish coding sequence. In contrast, the 5′ UTR-targeted Cyp1b1 MO is insensitive to both the zebrafish and human mRNA sequences (Supplementary Table S1). The 5′ UTR Cyp1b1 MO was used in *cyp1b1* mRNA rescue experiments. In the current studies, both the ATG- and 5′ UTR-targeted Cyp1b1 MO showed similarity of phenotype.

Similarly, 2 nonoverlapping translation blocking Raldh2 MO were used to confirm the knockdown phenotype. The ATG-targeted Raldh2 MO, but not the 5′ UTR-targeted Raldh2 MO, overlapped with the zebrafish coding sequence (Supplementary Table S1). The 5′ UTR Raldh2 MO was used in *raldh2* mRNA rescue experiments. Morpholino oligonucleotide knockdown (ATG or 5′ UTR) decreased Raldh2 protein while *raldh2* mRNA overexpression increased Raldh2 protein detected by whole-mount immunostaining (Supplementary Figs. S1A–E).

The coding regions of zebrafish *cyp1b1* and *raldh2* genes and *GFP* were cloned into the pCS2p+ expression vector, and the sequences were verified. The human *CYP1B1* coding region was cloned into the pCRII-TOPO expression vector. Site-directed mutagenesis using Pfu Turbo (QuikChange Site-Directed Mutagenesis Kit; Stratagene, La Jolla, CA, USA) and custom-designed primers were used to introduce point mutations (Supplementary Table S2). Capped mRNA was synthesized (mMessage mMachine kit; Ambion Biosystems, Foster City, CA, USA) and resuspended at 50 to 150 ng/μL in nuclease-free water. Approximately 1 to 2 nL (50–300 pg total RNA) of zebrafish *cyp1b1* or *raldh2* mRNA or human *CYP1B1* (wild type or mutant) mRNA was coinjected with phenol red and *GFP* mRNA (50 ng/μL; 50–100 pg RNA) into one-cell-stage embryos.

For each experiment, ∼100 embryos were injected with MO and/or mRNA, and all experiments were replicated a minimum of three times. Embryos were counted, and phenotypes for developmental delay, abnormal craniofacial development, and coloboma were assessed at 72 and 96 hours post fertilization (hpf). The percentage of embryos with these phenotypes in each group was calculated. Morpholino- or mRNA-injected embryos were compared with control-injected embryos. Each experiment (of ∼100 embryos/group) was used as an experimental unit. The mean with standard error is shown in the text and tables. The data were statistically analyzed using ANOVA with post hoc Tukey pairwise tests, and *P* < 0.05 was considered statistically significant. Images shown are representative of all experiments.

### Pharmacologic Treatments

All-trans retinoic acid (RA; Sigma-Aldrich Corp., St. Louis, MO, USA), diethylaminobenzaldehyde (DEAB; Sigma-Aldrich Corp.), and phenylthiourea (PTU; Sigma-Aldrich Corp.) were diluted in dimethylsulfoxide (DMSO; Sigma-Aldrich Corp.) at a 1000× final concentration. The pharmacologic treatments were administered in the embryo media at final concentrations of 25 to 50 nM RA, 10 μM DEAB, and 0.003% PTU. Treatments were initiated between 3 and 24 hpf as indicated. The medium was changed every 24 hours as indicated. Approximately 50 embryos were included for each condition, and all experiments were replicated a minimum of three times. Embryos were counted and phenotypes were assessed as described above. Images shown are representative of all experiments.

### Imaging

Embryos were analyzed under a M205FA combi-scope (Leica Microsystems CMS GmbH, Wetzlar, Germany). Representative images of at least four embryos in each group per experiment were obtained using brightfield DFC290 (Leica Microsystems CMS GmbH) and/or fluorescent ORCA-ER (Hamamatsu, Hamamatsu City, Japan) cameras. Sections were imaged under a DM6000B upright microscope (Leica Microsystems CMS GmbH) equipped with a DFC500 camera (Leica Microsystems CMS GmbH). The images were processed and analyzed using Adobe Photoshop (San Jose, CA, USA), LAS software (Leica Microsystems CMS GmbH), and/or AF6000 software (Leica Microsystems CMS GmbH).

Time-lapse imaging of Tg(*foxd3:GFP*) embryos was performed as previously described.^23^ Briefly, the embryos were imaged using a TCS SP5 MP multiphoton microscope (Leica Microsystems CMS GmbH) fitted with a Mai Tai DeepSee Ti-Sapphire laser (Spectra-Physics; Newport Corp., Irvine, CA, USA). The images were obtained every 20 minutes during the described time frame. Each time-lapse experiment was repeated three or four times. Images and movies included are representative of all experiments.

### Immunostaining and TUNEL Assay

Staged zebrafish embryos were fixed in 4% paraformaldehyde overnight at 4°C. For sectioning, the embryos were cryoprotected in successive sucrose solutions, embedded in optical cutting temperature (OCT) compound, and subsequently sectioned at 10 μm.

Whole-mount immunostaining was performed as previously described using primary rabbit anti-laminin (1:200; Sigma-Aldrich Corp.), primary rabbit anti-Raldh2 (1:100; GeneTex, Irvine, CA, USA), and secondary goat anti-rabbit IgG conjugated to Cy3 or 488 (1:1000; Abcam, Cambridge, MA, USA).^11^ Section immunostaining for GFP was performed using mouse anti-GFP directly conjugated to FITC (1:100; EMD Millipore, Billerica, MA, USA).

The percentage of embryos with continuous laminin staining in the distal ocular fissure was calculated. Morpholino- or mRNA-injected embryos were compared with control-injected embryos. The experiments were repeated three times, and each experiment (of ∼20 embryos/group) was used as an experimental unit. The mean with standard error is shown in the text. The data were statistically analyzed using ANOVA with post hoc Tukey pairwise tests, and *P* < 0.05 was considered statistically significant. Images shown are representative of all experiments.

The terminal deoxynucleotidyl transferase (dUTP) nick-end labeling (TUNEL) assay was performed as previously described using standard protocols.^11^ Briefly, apoptotic cells were detected through the TdT-mediated incorporation of digoxigenin-labeled deoxyuridine triphosphate (Roche Life Sciences, Indianapolis, IN, USA). Sheep anti-digoxigenin conjugated to rhodamine (Roche Life Sciences) was used to detect the TUNEL signal. The sections were costained with 4′,6-diamidino-2-phenylindole (DAPI). For quantification, three to five consecutive sections through the equator of the lens of at least four embryos per group were counted. The total number of cell nuclei and TUNEL-positive cells in the eyes of each section were manually counted. The percentage of TUNEL-positive cells in the eyes of MO- or mRNA-injected embryos was compared with those of control-injected embryos. The data were statistically analyzed using ANOVA with post hoc Tukey pairwise tests, and *P* < 0.05 was considered statistically significant. Images shown are representative of all experiments.

### In Situ Hybridization

In situ hybridization was performed through standard protocols using digoxigenin- or fluorescein-labeled RNA antisense probes.^17,25^ The *cyp1b1* RNA probe was 1047 base pairs, the *vsx1* RNA probe was 797 base pairs, the *vsx2* RNA probe was 503 base pairs, the *shha* RNA probe was 911 base pairs, the *shhb* RNA probe was 876 base pairs, the *pax6a* RNA probe was 458 base pairs, and the *pax6b* RNA probe was 316 base pairs. All probes were hybridized at 56°C overnight. For colorimetric reactions and signal comparisons, the embryos were developed for equal amounts of time. Sense controls were also developed in parallel to ensure specific staining. The embryos were cryoprotected, embedded, and sectioned at 10 μm. The sections were washed in phosphate-buffered saline, coverslipped, and imaged as described above.

### Western Blotting

Embryos were injected as described above, and total protein was isolated at 24 hpf. Twenty micrograms of protein was combined with sample buffer (10% glycerol, 62.5 mM Tris base, pH 6.8, 2.5% sodium dodecyl sulfate, 5% β-mercaptoethanol, and 0.002% bromophenol blue), boiled for 5 minutes, and loaded onto a 12% acrylamide gel (BioRad, Hercules, CA, USA). Protein was transferred to a nitrocellulose membrane (Life Technologies, Waltham, MA, USA), blocked in PBS/0.1% Tween–20/3% nonfat dry milk, and subsequently incubated with rabbit polyclonal anti-CYP1B1 (1:500, Ab157578, Ab78044; Abcam), mouse monoclonal anti-β-actin (1:1000, Ab8226; Abcam) overnight at 4°C. The membrane was washed in PBS/0.1% Tween-20 and subsequently incubated with antirabbit horseradish peroxidase (HRP)-conjugated secondary antibody (1:1000; Cell Signaling Technology, Danvers, MA, USA) or anti-mouse-HRP conjugated secondary antibody (1:1000, Cell Signaling Technology). Pierce ECL Western Blotting Substrate (Thermo Scientific, Waltham, MA, USA) was used for chemiluminescent detection. Commercially available antibodies (Abcam Ab157578 and Ab78044) against human CYP1B1 (∼61 kDa) did not show cross-reactivity with the zebrafish form (∼60 kDa) by Western blotting (see Fig. 7).

**Figure 1:**
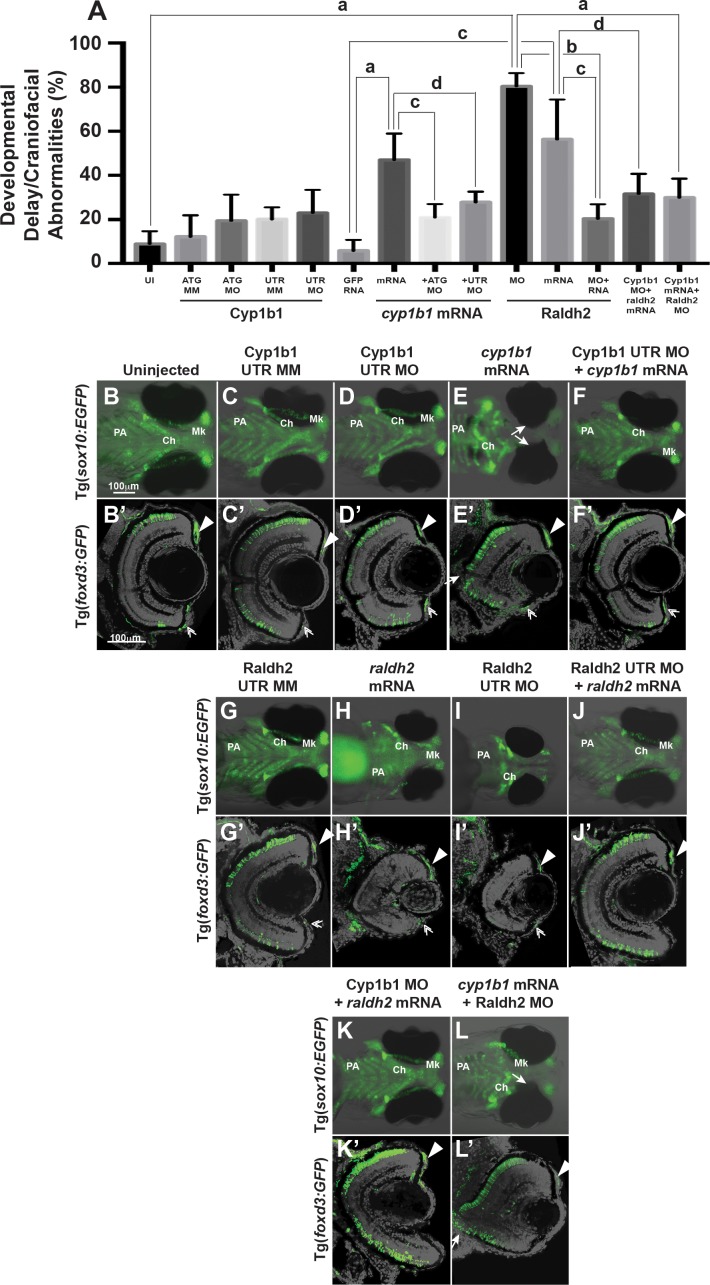
Overexpression of *cyp1b1* inhibited ocular fissure closure via an RA-independent pathway. The percentage of 72 hpf embryos with developmental delay and/or craniofacial abnormalities (**A**) due to Cyp1b1 MO (ATG or 5′ UTR) knockdown, *cyp1b1* (mRNA) overexpression, Raldh2 MO knockdown, *raldh2* (mRNA) overexpression compared to uninjected, mismatch (MM) MO-injected or *gfp* mRNA-injected controls. ([**a**] *P* < 0.0001; [**b**] *P* < 0.001; [**c**] *P* < 0.01; [**d**] *P* < 0.05). Live images of uninjected (**B**) and mismatch control-injected (**C**, **G**) Tg(*sox10:EGFP*) embryos showed that at 96 hpf, neural crest contributed to the pharyngeal arches (PA) and the ceratohyal (Ch) and Meckel's (Mk) cartilage of the jaw. Sections of 96 hpf Tg(*foxd3:GFP*) embryos showed that *foxd3*-positive neural crest cells contributed to the iris stroma in the dorsal (**B'**, **C'**, **G'**, *arrowhead*) and ventral (*double arrowhead*) iridocorneal angle. In addition, differentiated retinal photoreceptors expressed *foxd3*, which was unrelated to neural crest cells. Injection of 5′ UTR Cyp1b1 MO had minimal effect on craniofacial neural crest (**D**) and overall eye (**D'**) development. Overexpression of *cyp1b1* through mRNA injection disrupted neural crest–derived Meckel's cartilage formation (**E**, **K**) and inhibited ocular fissure closure, resulting in prominent colobomas (*closed arrows*, **E**, **E'**). The retinal layers were properly differentiated, including the expression of *foxd3*. Injection of Cyp1b1 5′ UTR MO improved the ocular and neural crest phenotypes due to *cyp1b1* mRNA (**F**, **F'**). As previously demonstrated, increasing (*raldh2* mRNA, **H**, **H'**) or decreasing (Raldh2 5′ UTR MO, **I**, **I'**) RA inhibited pharyngeal arch development, disrupted ceratohyal and Meckel's cartilage formation, and decreased eye size. Injection of *raldh2* mRNA rescued the ocular and neural crest phenotypes induced through Raldh2 5′ UTR MO knockdown (**J**, **J'**). Cyp1b1 MO improved eye size, retinal differentiation, and neural crest–derived pharyngeal arch and ceratohyal and Meckel's cartilage formation in embryos injected with *raldh2* mRNA (**K**, **K'**). Knockdown of Raldh2 improved neural crest–derived pharyngeal arch and ceratohyal and Meckel's cartilage formation (**L'**), but did not rescue ocular fissure closure (*arrows*, **L'**) in embryos injected with *cyp1b1* mRNA.

**Figure 2:**
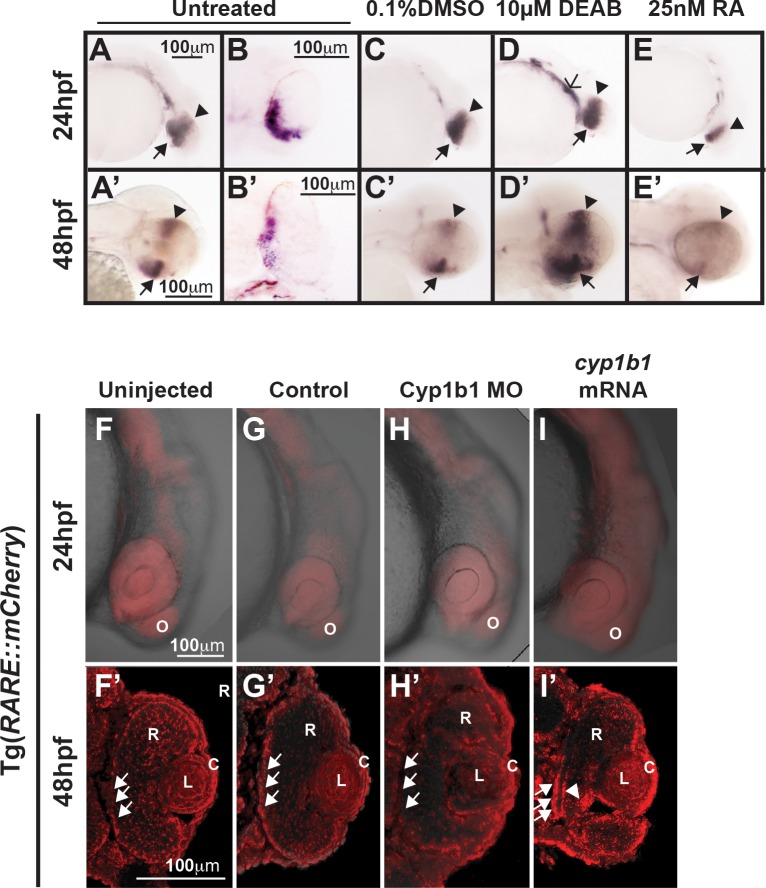
*Cyp1b1* expression in the developing eye was regulated through RA. Whole-mount in situ hybridization showed *cyp1b1* expression along the dorsal (*arrowheads*)–ventral (*arrows*) axis in the developing eye at 24 and 48 hpf in uninjected (**A**, **A'**) and 0.1% DMSO-treated (**C**, **C'**) embryos. In sections, *cyp1b1* localized to the retina and retinal pigment epithelium (**B**, **B'**). Treatment with 10 μM DEAB at 12 hpf increased *cyp1b1* expression in the ventral neural crest, which subsequently forms the brachial arches (**D**, *open arrowhead*) at 24 hpf and the dorsal (*arrowheads*) and ventral (*arrows*) eye at 48 hpf (**D'**). Exogenous treatment with 25 nM all-trans RA at 12 hpf decreased *cyp1b1* expression in the dorsal and ventral eye at 24 (**E**) and 48 hpf (**E'**). Live imaging of the Tg(*RARE:mCherry*) reporter line showed areas of high RA activity in the eye and olfactory placode (O) in uninjected (**F**) and control-injected (**G**) embryos. Within the eye (**F'**, **G'**), RA activity was observed in the retinal pigment epithelium (*arrows*), retina (R), lens (L), and cornea (C). MO knockdown of Cyp1b1 (**H**, **H'**) showed decreased RA activity in the medial retinal pigment epithelium (*arrows*) correlated with the area of *cyp1b1* expression (**B'**). Overexpression of *cyp1b1* through mRNA injection diffusely increased mCherry expression at 24 hpf throughout the craniofacial region (**I**). Increased RA activity in the eye was also observed in the medial retinal pigment epithelium (*arrows*) and ocular fissure (*arrowhead*).

**Figure 3:**
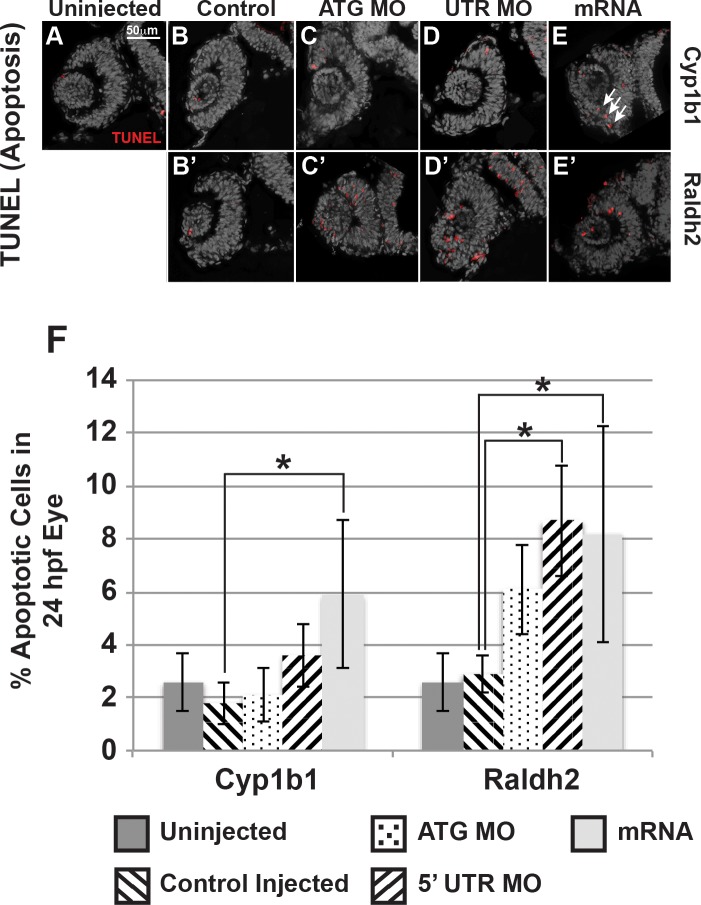
Cyp1b1 and RA regulated cell survival in the developing eye. Cyp1b1 ATG (**C**) and 5′ UTR (**E**) MO knockdown did not significantly affect apoptosis in the eye at 24 hpf compared to uninjected (**A**) and control-injected (**B**) embryos. *Cyp1b1* overexpression (**E**) increased apoptosis and the apoptotic cells were specifically localized to the inferior ocular fissure (*arrows*). Raldh2 5′ UTR (**D'**) but not ATG (**C'**) MO knockdown significantly decreased cell survival throughout the developing eye compared to uninjected (**A**) and control injected (**B'**). Similarly, overexpression of *raldh2* (**E'**) increased apoptosis in the eye. The percentage of apoptotic cells in 24 hpf developing eyes (**F**) of embryos injected with Cyp1b1 MO (ATG or 5′ UTR), *cyp1b1* mRNA, Raldh2 (ATG or 5′ UTR) MO, *raldh2* mRNA compared to uninjected and control injected (*P < 0.01).

**Figure 4:**
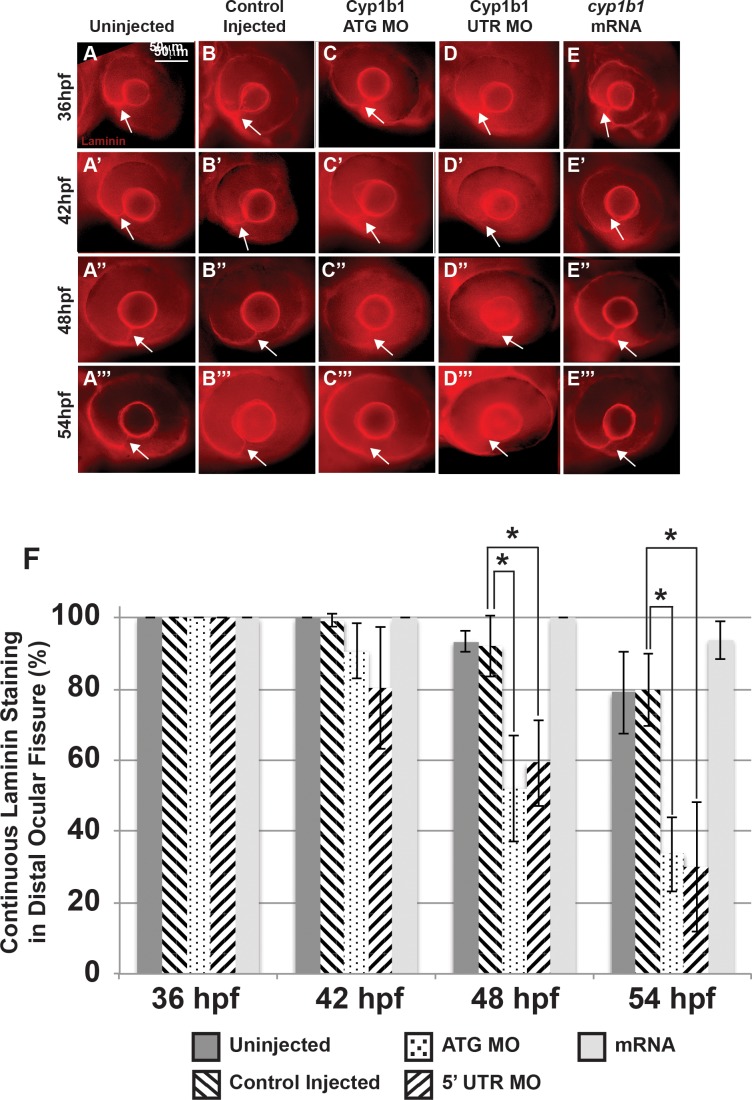
Alterations in the basement membrane integrity as a result of decreased or increased Cyp1b1. Whole-mount laminin staining showed that in uninjected (**A**–**A'''**) and control-injected (**B**–**B'''**) embryos, a continuous basement membrane (*arrows*) was evident in the distal ocular fissure at 36, 42, 48, and 54 hpf. Cyp1b1 ATG (**C**–**C'''**) and 5′ UTR (**D**–**D'''**) MO knockdown showed discontinuous staining of laminin at 48 (**C''**, **D''**) and 54 hpf (**C'''**, **D'''**), suggesting premature basement membrane breakdown. In *cyp1b1* mRNA-injected embryos, laminin staining remained prominent within the fissure at all time points (**E**–**E'''**). The percentage of embryos with continuous laminin staining in the distal ocular fissure at 36, 42, 48, and 54 hpf (**G**), (*P < 0.001).

**Figure 5:**
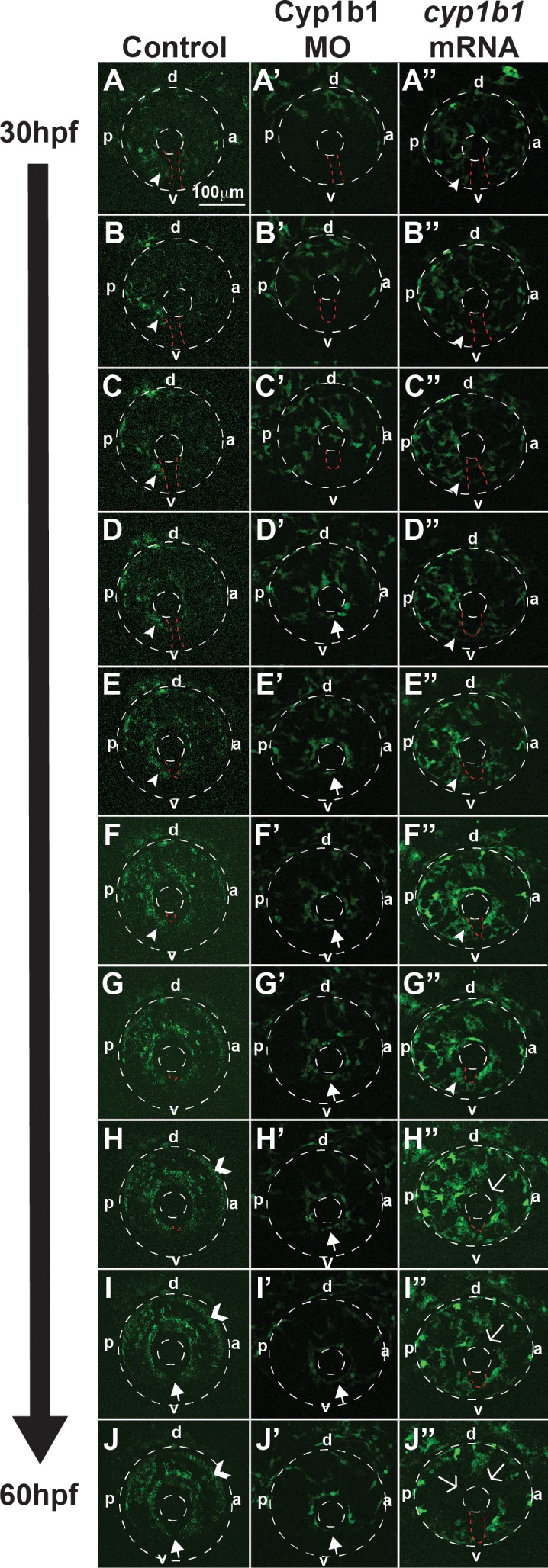
*Cyp1b1* regulates ocular neural crest migration. Time-lapse imaging of Tg(*foxd3:EGFP*) control embryos from 30 to 60 hpf (**A**–**J**) showed that *foxd3*-positive cells entered into the anterior chamber between the surface ectoderm and optic cup, with more cells localized to the dorsal (d)–posterior (p) quadrant (**E**–**H**) compared with the anterior (a) and ventral (v) areas. In addition, *foxd3*-positive cells migrated adjacent to and through the ocular fissure (**A**–**F**, *white arrowhead* denotes migrating neural crest, *red dashed line* demarcates ocular fissure). At 60 hpf, *foxd3*-positive cells completely encircled the lens (**I**, **J**, *closed arrows*), indicating the closure of the fissure. In addition, at 60 hpf, *foxd3* was also expressed in photoreceptors (**H**–**J**, *open arrowhead*). In Cyp1b1 MO knockdown embryos, *Foxd3*-positive cells travelled between the surface ectoderm and optic cup in the dorsal quadrants (**A'**–**E'**), but there were few cells adjacent to and in the ocular fissure. *Foxd3*-positive cells encircled the lens prior to controls (**D'**–**H'** versus **D**–**H**), indicating the premature closure of the fissure. At 60 hpf, there were fewer *foxd3*-positive cells in the eye (**J'**). In embryos overexpressing *cyp1b1*, ocular neural crest migration was less organized, as *foxd3*-positive cells were located throughout the posterior half of the eye (**A''**–**G''**). Further, the ocular fissure remained open and prevented the continuous coalescence of neural crest cells around the lens (**H''**–**J''**, *open arrows*), resulting in fewer neural crest cells at 60 hpf (**J''**).

**Figure 6:**
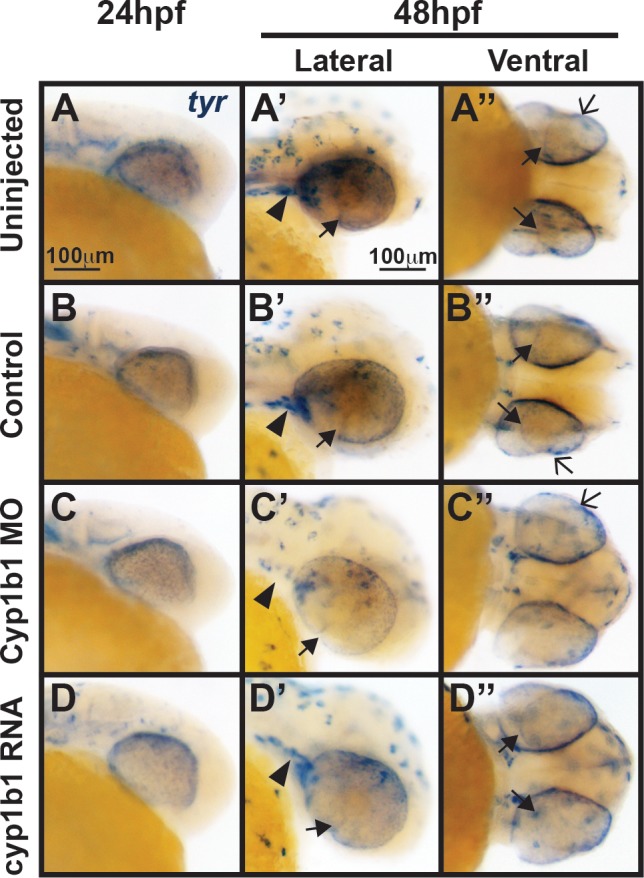
*Tyr* expression is not influenced by alterations in *cyp1b1* expression. In situ hybridization demonstrated that at 24 hpf, *tyr* was expressed in the migrating neural crest and retinal pigment epithelium in uninjected (**A**) and control-injected (**B**) embryos. At 48 hpf, *tyr* expression (**A'**, **A''**, **B'**, **B''**) followed the skin pigmentation pattern and was also observed in the cranial neural crest (*arrowheads*), retinal pigment epithelium, migrating neural crest cells in the ocular fissure (*closed arrows*), and iris stroma (*open arrows*). Knockdown of *cyp1b1* did not alter *tyr* expression at 24 hpf, but did decrease *tyr* expression in the cranial neural crest (*arrowhead*) and ocular fissure (**C**, **C'**) at 48 hpf. Overexpression of *cyp1b1* did not alter *tyr* expression (**D**, **D'**, **D''**) at 24 and 48 hpf.

**Figure 7:**
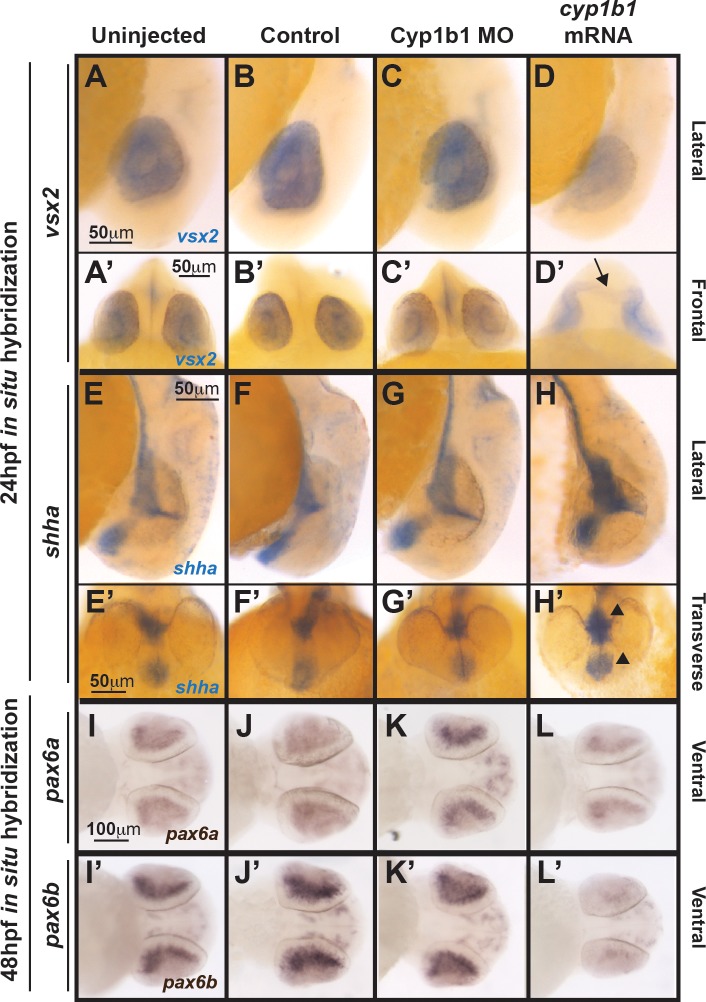
*Cyp1b1* differentially regulated *vsx2*, *shh*, and *pax6*. Whole-mount in situ hybridization at 24 hpf demonstrated that *vsx2* expression in the eye decreased in response to *cyp1b1* overexpression (**D**, **D'**) compared with Cyp1b1 MO knockdown (**C**, **C'**), control-injected (**B**, **B'**), and uninjected (**A**, **A''**) embryos. Note the appearance of tissue bridging the two optic cups in the *cyp1b1* mRNA-injected embryo, as demarcated by *vsx2* (*arrow*). *Shha* at 24 hpf showed increased expression in the midline floor plate between the eyes in embryos injected with *cyp1b1* mRNA (*arrowheads*, **H**, **H'**) compared with Cyp1b1 MO knockdown (**G**, **G'**), control -injected (**F**, **F'**), and uninjected (**E**, **D'**) embryos. *Pax6a* and *pax6b* expression in the retina at 48 hpf decreased in response to *cypb1* overexpression (**L**, **L'**) compared with Cyp1b1 knockdown (**K**, **K'**), control-injected (**J**, **J'**), and uninjected (**I**, **I'**) embryos.

### Methylacrylate Sections

Zebrafish embryos at 96 hpf were fixed in 2% paraformaldehyde/1.5% glutaraldehyde overnight at 4°C, followed by embedding in methylacrylate. The blocks were sectioned at 5 μm. The sections were stained with Lee's stain, coverslipped, and subsequently imaged as described above.

## Results

### *Cyp1b1* Overexpression Inhibited Ocular Fissure Closure

Previously published studies have established an effective MO that blocks Cyp1b1 translation; however, these studies did not assess eye or craniofacial development.^24^ Because mutations in *CYP1B1* are associated with primary infantile-onset glaucoma, reflecting abnormalities in the neural crest–derived iridocorneal angle, the Tg(*sox10:EGFP*) and Tg(*foxd3:GFP*) transgenic zebrafish strains in which GFP is expressed in different subpopulations of neural crest cells were used to analyze neural crest and eye development.

Two different translation blocking Cyp1b1 MOs (ATG or 5′ UTR) were used to determine the effects of knockdown on neural crest and eye development and verify consistency of phenotype. At 72 hpf, the most common phenotypes in Cyp1b1 ATG and 5′ UTR MO knockdown embryos included developmental delays of 12 to 24 hours and craniofacial abnormalities in 20.5 ± 12.1% and 26.8 ± 11.1% of embryos, respectively (Fig. 1A; Supplementary Table S3). This finding was not significantly different compared with the percentage of developmentally delayed and abnormal Cyp1b1 ATG mismatch MO injected (12.1 ± 9.8%, *P* = 0.56) or Cyp1b1 5′ UTR mismatch MO injected (21.0 ± 7.1%, *P* = 0.97). Further, there were no significant differences between mismatch control-injected embryos and uninjected (8.3% ± 6.3%, *P* = 0.98, ATG mismatch, and *P* = 0.10, 5′ UTR mismatch) embryos.

At 96 hpf, MO knockdown of Cyp1b1 had a minimal effect on craniofacial neural crest development, including the pharyngeal arches (PA), ceratohyal cartilage (Ch), and Meckel's (Mk) cartilage (Fig. 1D; Supplementary Fig. S2B) compared with controls (Fig. 1C; Supplementary Fig. S2A) and uninjected (Fig. 1B) embryos. Although we previously showed that Cyp1b1 knockdown delayed eye development at 48 hpf,^26^ by 96 hpf, anterior segment and retinal development (Fig. 1D'; Supplementary Fig. S2B') had caught up to control-injected (Fig. 1C'; Supplementary Fig. S2A') and uninjected (Fig. 1B') embryos. This included *foxd3*-positive neural crest cells in the dorsal (arrowheads) and ventral (double arrowheads) iris stroma. In addition, *foxd3* was expressed in differentiated retinal photoreceptors, which was unrelated to expression of this transcription factor in neural crest cells.

In contrast, the injection of *cyp1b1* mRNA markedly affected craniofacial and eye development. At 72 hpf, a significant percentage (43.8 ± 12.1%, *P* < 0.0001) of *cyp1b1* mRNA-injected embryos showed developmental delays and craniofacial abnormalities compared with *gfp* mRNA-injected (5.7 ± 4.9%) and uninjected embryos (Fig. 1A; Supplementary Table S3). Furthermore, at 72 hpf, 38.7 ± 8.5% (*P* = 0.0001) of *cyp1b1* mRNA-injected embryos showed ocular fissure closure defects (delayed closure and coloboma), which were not observed in *gfp*-injected or uninjected embryos. At 96 hpf, *cyp1b1* overexpression inhibited Mk cartilage development (Fig. 1E) and caused eye wall defects and colobomas (Figs. 1E, 1E', arrows). Although loss of the retinal pigment epithelial layer was observed in the area of the coloboma, the retinal layers were properly differentiated. In addition, *foxd3*-positive cells were observed in the neural crest–derived iris stroma (arrowheads and double arrowheads). The injection of Cyp1b1 ATG or 5′ UTR MO significantly decreased the percentage of embryos (20.9 ± 6.0%, *P* = 0.002 and 27.9 ± 4.8%, *P* = 0.03) with ocular and craniofacial defects due to *cyp1b1* mRNA injection (Supplementary Table S3; Figs. 1A, 1F, 1F'). Although decreased *cyp1b1* caused only mild developmental delays and abnormalities, the overexpression of *cyp1b1* demonstrated that this gene plays a role in ocular fissure closure and cranial neural crest development.

### *Cyp1b1* Expression in the Ocular Fissure Was Regulated Through RA

The role of *cyp1b1* in eye development has been hypothesized to involve RA, as c*yp1b1* regulates RA synthesis in vitro.^5^ We determined whether *cyp1b1* expression was regulated through alterations in RA levels. In situ hybridization revealed that *cyp1b1* was expressed in the dorsal (arrowhead) and ventral (closed arrow) retina and retinal pigment epithelium at 24 (Figs. 2A, 2B, 2C) and 48 hpf (Figs. 2A', 2B', 2C') in untreated and DMSO-control treated embryos. *Cyp1b1* expression was spatially and temporally correlated with the superior and inferior ocular fissures. Diethylaminobenzaldehyde is a pan-aldehyde dehydrogenase inhibitor that decreases endogenous RA synthesis mediated by *raldh2* and *raldh3*. Treatment at 12 hpf with 10 μM DEAB did not alter *cyp1b1* expression in the developing eye at 24 hpf (Fig. 2D), but did increase *cyp1b1* expression in the ventral migrating neural crest (open arrowhead) compared with DMSO-treated control (Fig. 2C) and untreated embryos (Fig. 2A). At 48 hpf, DEAB increased *cyp1b1* expression in the dorsal (arrowhead) and ventral (closed arrow) eye (Fig. 2D') compared with controls (Figs. 2A', 2C'). In contrast, treatment with exogenous RA (25 nM) at 12 hpf inhibited *cyp1b1* expression in the eye at 24 (Fig. 2E) and 48 hpf (Fig. 2E'). Thus, *cyp1b1* expression was affected by pharmacologic alterations in RA levels.

To determine whether the converse was also true, a transgenic line (Tg[*RARE:mCHERRY*]), in which mCherry expression is driven by canonical RA response elements (RARE), was utilized. At 24 hpf, RA expression in the craniofacial region was greatest in the eye and the olfactory placode in uninjected and control-injected embryos (Figs. 2F, 2G; Supplementary Figs. S3A, S3B). At 48 hpf, within the eye, diffuse RA activity was observed throughout the retina, lens, and cornea (Fig. 3F'). Cyp1b1 MO knockdown did not show alterations in RA activity in the developing head at the doses tested (Fig. 2H; Supplementary Fig. S3C). In the eye, decreased RA activity was observed in the retinal pigment epithelium (Fig. 3H') in the area of *cyp1b1* expression (Fig. 3B') compared with control-injected (Fig. 3G') and uninjected (Fig. 3F') embryos. In contrast, the overexpression of *cyp1b1* by injecting mRNA at the one-cell stage caused a diffuse increase in mCherry expression throughout the craniofacial region (Fig. 3I; Supplementary Fig. S3D). Further, in the eye, increased RA activity was observed in the retinal pigment epithelium and retina (Fig. 3I', arrows) and in the region of the ocular fissure (arrowhead) compared with the eyes of control-injected (Fig. 3G') and uninjected (Fig. 3F') embryos. These studies suggest that *cyp1b1* expression was regulated by RA, and the overexpression of *cyp1b1* may alter local RA levels in and around the developing eye.

### *Cyp1b1* Regulation of Ocular Fissure Closure Was Independent of RA

Since *cyp1b1* expression was regulated through RA, we assessed whether *cyp1b1* and *raldh2* worked together to control eye and neural crest development. As previously demonstrated, *raldh2* overexpression (Figs. 1H, 1H') and Raldh2 MO knockdown (Figs. 1I, 1I'; Supplementary Figs. S2C, S2C') significantly decreased eye size and inhibited neural crest–derived jaw and PA formation in 56.2 ± 17.7% (*P* < 0.002) and 80.4 ± 5.9% (*P* = 0.0001) of injected embryos, respectively (Fig. 1A; Supplementary Table S3).^10^ The coinjection of Raldh2 MO and *raldh2* mRNA significantly decreased the percentage of embryos with craniofacial and ocular defects (20.5 ± 6.8%, *P* = 0.01), demonstrating the specificity of these reagents (Supplementary Table S3; Figs. 1A, 1H, 1H'). To determine whether the Cyp1b1-mediated regulation of neural crest and eye development reflects the regulation of RA synthesis, Cyp1b1 MO was coinjected with *raldh2* mRNA. At 72 hpf, Cyp1b1 knockdown decreased the percentage of embryos with small eyes and craniofacial defects (31.4 ± 9.0%, *P* = 0.04, Fig. 1A; Supplementary Table S3) resulting from the overexpression of *raldh2*. At 96 hpf, Cyp1b1 knockdown with *raldh2* overexpression improved eye size and craniofacial cartilage formation (Figs. 1K, 1K'; Supplementary Figs. S2D, S2D'). *Cyp1b1* overexpression decreased the percentage of 72 hpf Raldh2 MO knockdown embryos with developmental delay, small, malformed eyes and craniofacial abnormalities (53.5 ± 7.7%, *P* = 0.0001, Fig. 1A; Supplementary Table S3). At 96 hpf, the overexpression of *cyp1b1* improved eye size and jaw and PA development in Raldh2 MO knockdown embryos (Figs. 1L, 1L'; Supplementary Figs. S2E, S2E'). However, decreased RA through Raldh2 MO knockdown did not decrease the percentage of *cyp1b1* mRNA-injected embryos with ocular fissure defects at 72 hpf (30.0 ± 8.7%, *P* = 0.17, Supplementary Table S3) and 96 hpf (Fig. 1L', Supplementary Fig. S2E').

This finding was further confirmed using a DEAB dose–response curve (1–5 μM) in combination with *cyp1b1* overexpression (Supplementary Fig. S4; Supplementary Table S4). Treatment of *GFP* mRNA-injected embryos with 1 μM DEAB (at 3 hpf) did not significantly affect ocular fissure closure (28.0 ± 7.0%, *P* = 0.10) compared with untreated GFP-injected controls (3.3 ± 1.0%). However, treatment with 2 or 5 μM DEAB significantly increased the percentage of embryos (38.0 ± 9.4% [*P* = 0.008] and 55.2 ± 4.6% [*P* < 0.0001], respectively) with ocular closure defects at 72 hpf. At 96 hpf in GFP-injected control embryos, increasing concentrations of DEAB (Supplementary Figs. S4E, S4G, S4I) altered PA and Ch and Mk cartilage formation, decreased eye size, and delayed ocular fissure closure (arrowhead) compared with untreated (Supplementary Fig. S4A) and DMSO-control treated (Supplementary Fig. S4C) embryos. Treatment with DEAB did not improve or rescue the ocular fissure defects in *cyp1b1* mRNA-injected embryos, but rather significantly increased the percentage of embryos with defects at each concentration (82.2 ± 13.4% at 1 μM DEAB [*P* = 0.0007], 84.5 ± 14.4% at 2 μM DEAB [*P* = 0.0003], and 100% at 5 μM DEAB [*P* < 0.0001]). *Cyp1b1* mRNA-injected embryos treated with 1, 2, or 5 μM DEAB showed open ocular fissures (arrowheads), ocular wall defects (arrows), small eyes, and jaw and PA defects (Supplementary Figs. S4F, S4H, S4J). Concentrations of DEAB higher than 5 μM were lethal in both GFP- and *cyp1b1* mRNA-injected embryos (data not shown). Thus, the effect of *cyp1b1* overexpression on ocular fissure closure was not mediated through RA.

Next, the effect of *cyp1b1* on cell survival in the developing eye, particularly in the ocular fissure, was assessed. At 24 hpf, TUNEL assay of the eyes of embryos injected with *cyp1b1* mRNA (5.9 ± 2.8%, Figs. 3E, 3F) showed a statistically significant (*P* = 0.005) 2-fold increase in apoptosis compared with uninjected (2.6 ± 1.1%, Fig. 2A) and control-injected embryos (1.8 ± 0.8% Fig. 3B). Many of the apoptotic cells were localized to the inferior ocular fissure (arrows). In contrast, injection of Cyp1b1 ATG (2.1 ± 1.0%, Fig. 2C) or 5′ UTR MO (3.6 ± 1.2%, Fig. 3D) did not significantly affect apoptosis. *Raldh2* overexpression (8.2 ± 4.1%, Fig. 3E') significantly (*P* = 0.003) increased the percentage of apoptotic cells in the developing eye compared to uninjected (Fig. 3A) or control-injected embryos (2.9 ± 0.7%, Fig. 3B'). Injection of Raldh2 5′ UTR MO (8.7 ± 2.1%, Fig. 3D'), but not ATG MO (6.1 ± 1.7%, Fig. 3C'), significantly (*P* = 0.005) increased apoptosis. However in both the *raldh2* overexpression and Raldh2 knockdown embryos, apoptotic cells were distributed throughout the eye and were not specifically localized to the ocular fissure. Thus, Cyp1b1 increased apoptosis in the ventral ocular fissure and regulated fissure closure via an RA-independent pathway.

### Cyp1b1 Maintained the Patency of the Inferior Ocular Fissure

Ocular fissure closure is a complex process, requiring optic vesicle evagination, spatial specification along the proximal–distal and ventral–dorsal axes, breakdown of the basement membrane that demarcates the fissure, and fusion of the two sides (reviewed in Refs. 27, 28). The temporal and spatial expression of *cyp1b1* in the ocular fissure and the colobomatous defects resulting from *cyp1b1* overexpression suggested that *cyp1b1* plays a role in fissure closure. We used whole-mount laminin staining to examine basement membrane integrity in the ocular fissure as a measure for the timing of ocular fissure closure. In uninjected (Figs. 4A–A''') and control-injected (Figs. 4B–B''') embryos, laminin staining was observed in the distal ocular fissure (arrows) between 36 and 54 hpf. In uninjected embryos, laminin staining was found in a continuous line indicating presence of the basement membrane between the two sides of the ocular fissure in 100% of 36 and 42 hpf embryos, 93.4 ± 2.9% of 48 hpf embryos, and 79.1 ± 11.7% of 54 hpf embryos (Fig. 4F). Similarly, control-injected embryos showed laminin staining in 100% of 36 hpf embryos, 99.3 ± 1.7% of 42 hpf embryos, 92.1 ± 8.8% of 48 hpf embryos, and 79.8 ± 8.8% of 54 hpf embryos, (Fig. 4G). Injection of Cyp1b1 ATG (Figs. 4C–C''') or 5′ UTR (Figs. 4D–D''') MO caused early breakdown of the basement membrane in the ocular fissure. At 42 hpf, injection of Cyp1b1 ATG or 5′ UTR MO did not significantly decrease the percentage of embryos with continuous laminin staining in the fissure (90.8 ± 8.0% and 80.4 ± 17.2%, respectively). However, injection of Cyp1b1 ATG or 5′ UTR MO significantly decreased the percentage of embryos with continuous laminin staining in the ocular fissure at 48 hpf (52.2 ± 15.0%, *P* = 0.002, and 59.3 ± 12.2%, *P* = 0.001) and 54 hpf (33.7 ± 10.5%, *P* = 0.0005, and 30.1 ± 18.3%, *P* = 0.0002). *Cyp1b1* overexpression (Figs. 4E', 4E''') showed laminin in the fissure in 100% of 36, 42, and 48 hpf and 93.9 ± 5.4% of 54 hpf embryos.

The neural crest migrates into the anterior segment of the eye, contributing to the cornea, iris, and iridocorneal angle. Because *cyp1b1* was expressed in the retina and retinal pigment epithelium and alterations in *cyp1b1* affected fissure closure, we used time-lapse imaging to investigate whether *cyp1b1* affected neural crest migration into the developing eye. Between 30 and 60 hpf, *foxd3*-positive neural crest cells [Tg(*foxd3:GFP*)] entered the anterior segment of the eye (Figs. 5A–J). *Foxd3*-positive cells migrated between the surface ectoderm and optic cup, with the majority of cells localized to the dorsal–posterior quadrant (Figs. 5E–H, dorsal denoted with “d”, ventral denoted with “v”, anterior denoted with “a” and posterior denoted with “p”). Additional cells travelled adjacent to and through the ocular fissure (Figs. 5A–F; white arrowhead denotes migrating neural crest, red dashed line demarcates ocular fissure). At 60 hpf, *foxd3*-positive cells coalesced and organized to completely encircle the lens (Figs. 5I, 5J, closed arrows), indicating closure of the fissure. In addition, at 60 hpf, *foxd3* was also expressed in the photoreceptors (Figs. 5H–J, open arrowhead). Cyp1b1 knockdown altered *foxd3*-positive cell migration between 30 and 60 hpf (Figs. 5A'–J'). *Foxd3*-positive cells travelled between the surface ectoderm and optic cup in the dorsal quadrants (Figs. 5A'–E'), but poorly migrated in specific ventral quadrants (denoted “v”) adjacent to and through the ocular fissure (demarcated by red dashed line). *Foxd3*-positive cells encircled the lens earlier than in control embryos (Figs. 5D'–H' versus Figs. 5D–H), suggesting premature closure of the fissure. At 60 hpf, fewer *foxd3*-positive cells were observed in the eyes of Cyp1b1 MO knockdown embryos (Fig. 5J'). In embryos in which *cyp1b1* was overexpressed, ocular neural crest migration was less organized, as *foxd3*-positive cells were located throughout the posterior half of the eye (Figs. 5A''–G''). Further, the ocular fissure (demarcated by red dashed lines) remained open, preventing the continuous coalescence of neural crest cells around the lens (Figs. 5H''–J'', open arrows). Thus, the mis-expression of *cyp1b1* altered ocular neural crest migration and subsequent iris stromal organization.

### Tyrosinase Did Not Modify the Effect of Cyp1b1 in Zebrafish Eye Development

Previously published studies in mice have demonstrated that tyrosinase modifies the effect of *Cyp1b1* knockout; however, the specific interactions between these two enzymes in neural crest and anterior segment development remain unknown.^4^ This relationship was further investigated in zebrafish embryos. In 24 hpf wild-type zebrafish embryos, the tyrosinase gene (*tyr*) was expressed in the migrating neural crest and in the retinal pigment epithelium in uninjected and control-injected embryos (Figs. 6A, 6B). At 48 hpf, *tyr* expression (Figs. 6A', 6A'', 6B', 6B'') followed the skin pigmentation pattern and was also observed in the cranial neural crest (arrowheads), retinal pigment epithelium, migrating neural crest cells in the ocular fissure (closed arrows), and iris stroma (open arrows). At 48 hpf, Cyp1b1 knockdown decreased *tyr* expression in the cranial neural crest (arrowhead) and ocular fissure (Figs. 6C, 6C'). Overexpression of *cyp1b1* did not alter *tyr* expression (Figs. 6D, 6D', 6D'') at 24 and 48 hpf. Mutant mice deficient for both *Tyr* and *Cyp1b1* genes showed a worse anterior segment phenotype. We utilized pharmacologic and genetic strategies to decrease tyrosinase activity. Phenylthiourea is often used in zebrafish embryos to inhibit tyrosinase and subsequent pigmentation, but can cause developmental delays and abnormalities when administered prior to 22 hpf or at concentrations higher than 0.003%.^29^ To inhibit tyrosinase activity prior to *tyr* and *cyp1b1* expression, embryos were treated with 0.003% PTU between 10 and 12 hpf. Treatment of uninjected embryos with PTU did not significantly increase the percentage of embryos with developmental delays and craniofacial abnormalities (17.6 ± 7.0%, *P* = 0.99) compared with untreated/uninjected embryos. Compared with PTU-treated uninjected embryos, PTU did not cause a significant increase in developmental delays or abnormalities in control-injected (26.3 ± 3.5%, *P* = 0.28) or Cyp1b1 MO-injected embryos (29.0 ± 7.5%, *P* = 0.85). In addition, we utilized the *casper* mutant strain, which has decreased *tyr* expression in neural crest–derived cells. Injection of Cyp1b1 MO in *casper* embryos did not significantly increase the percentage of embryos with craniofacial abnormalities or developmental delays (25.0 ± 11.0%) compared to control-injected (19.4 ± 12.8%, *P* = 0.71) and uninjected *casper* embryos (8.4 ± 10.4 %). Hence, in zebrafish, alterations in tyrosinase expression and activity did not alter the Cyp1b1 effect on eye development.

### Cyp1b1 Differentially Regulated Genes Associated With Colobomas

Because Cyp1b1 regulation of ocular fissure closure was not mediated through RA, genes clinically associated with colobomas were investigated as downstream targets. In situ hybridization at 24 hpf demonstrated that *cyp1b1* overexpression inhibited the expression of the homeobox transcription factor *vsx2* in the developing eye (Figs. 7D, 7D') compared with Cyp1b1 MO knockdown (Figs. 7C, 7C'), control-injected (Figs. 7B, 7B'), and uninjected (Figs. 7A, 7A') embryos. Further, in situ hybridization revealed the presence of *vsx2*-expressing tissue bridging the area between the optic cups (Fig. 7D', arrow) in response to *cyp1b1* overexpression. In contrast, *cyp1b1* overexpression increased *shha* expression in the floor plate between the developing eyes (Figs. 7H, 7H', arrowheads) compared with Cyp1b1 MO knockdown (Figs. 7G, 7G), control-injected (Figs. 7F, 7F'), and uninjected (Figs. 7E, 7E') embryos. Alterations in *cyp1b1* levels did not affect the expression of *vsx1* and *shhb* (data not shown) at 24 hpf. Although the expression of *pax6a* and *pax6b* was not affected at 24 hpf (data not shown), the expression of these two *pax6* genes was decreased in the retina in response to *cyp1b1* overexpression at 48 hpf (Figs. 7L, 7L') compared with Cyp1b1 MO knockdown (Figs. 7K, 7K'), control-injected (Figs. 7J, 7J'), and uninjected (Figs. 7I, 7I') embryos. Thus, *cyp1b1* overexpression altered the expression of key signaling molecules involved in eye development and ocular fissure closure.

### Human *CYP1B1* and Zebrafish *cyp1b1* Genes Are Evolutionarily and Functionally Conserved

**Figure 8:**
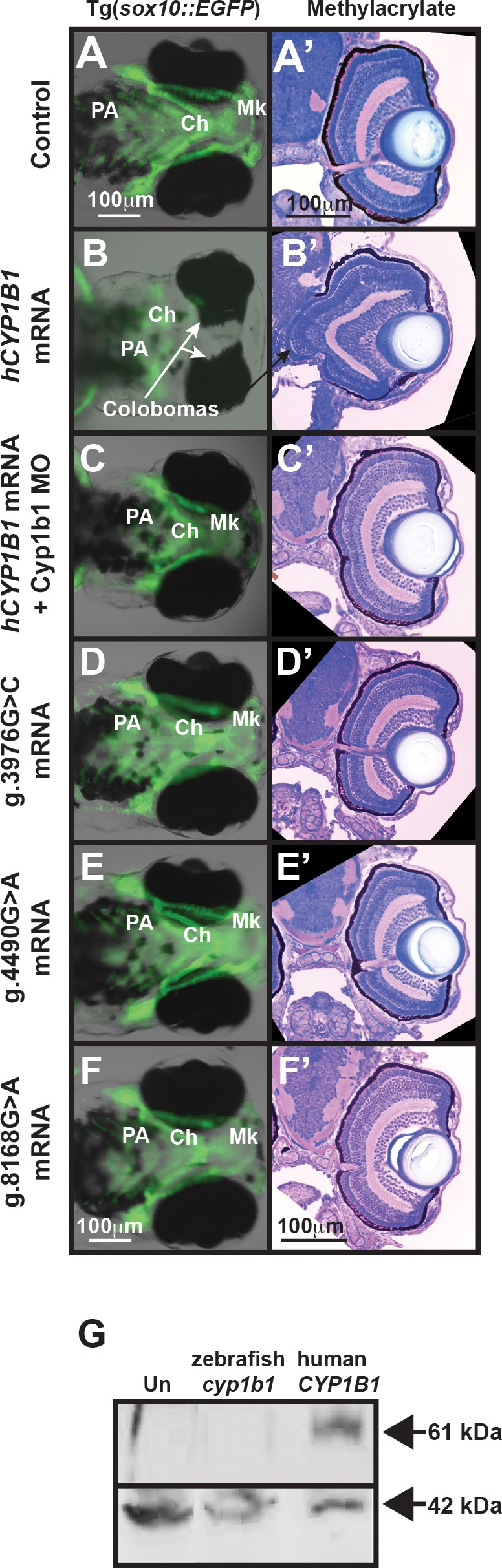
Human *CYP1B1* and zebrafish *cyp1b1* genes are evolutionarily and functionally conserved. Live images and methylacrylate sections of 96 hpf Tg(*sox10:EGFP*) embryos demonstrated that the injection of human *CYP1B1* mRNA disrupted neural crest–derived jaw and pharyngeal arch formation and inhibited ocular fissure closure (**B**, **B'**) compared with controls (**A**, **A'**). The knockdown of endogenous Cyp1b1 restored fissure closure (**C'**) and partially rescued the jaw and pharyngeal arch defects resulting from the injection of human *CYP1B1* mRNA (**C**). The injection of human *CYP1B1* mRNA containing clinically relevant g.3976G>C (**D**, **D'**), g.4490G>A (**E**, **E'**), or g.8168G>A (**F**, **F'**) mutations did not disrupt eye or neural crest development. Western blot analysis (**G**) showed expression of human CYP1B1 protein (61 kDa) in embryos injected with human *CYP1B1* mRNA sequence, but not in uninjected embryos or embryos injected with zebrafish *cyp1b1* mRNA. β-actin (42 kDa) was used as a loading control.

Mutations in CYP1B1 disrupt the development of the neural crest–derived trabecular meshwork and outflow channels and are the most commonly identified genetic causes of primary infantile-onset glaucoma.^3^ To investigate whether Cyp1b1 function is conserved between humans and zebrafish, human *CYP1B1* mRNA was injected into zebrafish embryos at the one-cell stage. Western blotting verified human *CYP1B1* protein expression in 24 hpf mRNA-injected embryos (Fig. 8G). Similar to the effects observed with the zebrafish gene, the overexpression of human *CYP1B1* caused large colobomas in 71.8 ± 17.9% of embryos (*P* = 0.0003, Supplementary Table S5), reflecting the defective closure of the ocular fissure (Figs. 8B, 8B') compared to controls (Figs. 8A, 8A'). Further, similar to the overexpression of zebrafish *cyp1b1*, human *CYP1B1* overexpression disrupted neural crest–derived jaw (Mk and Ch cartilage) and PA formation. The ATG MO-mediated knockdown of endogenous zebrafish Cyp1b1 showed improvement of craniofacial defects, but did not statistically significantly decrease the percentage of embryos with colobomas and craniofacial defects (36.4 ± 17.0, *P* = 0.06) due to human *CYP1B1* overexpression (Figs. 8C, 8C').

The genetic analysis of patients affected with primary infantile-onset glaucoma showed that disease-causing mutations typically occur in the second or third exon of the *CYP1B1* gene.^30^ Using site-directed mutagenesis, three mutations (genomic DNA nomenclature based on GenBank U56438) were introduced into the coding region of human *CYP1B1*, and the mRNAs for each of these mutants were transcribed and injected into one-cell embryos (Supplementary Table S2). The g.3976G>C mutation results in a tryptophan to cysteine (W57C) amino acid substitution that disrupts the hinge region connecting the N-terminal membrane-bound domain to the cytosolic portion of CYP1B1.^3,31^ The g.4490G>A mutation results in a glutamic acid to lysine (E229K) amino acid substitution that alters the ionic bonds necessary for substrate binding.^3,32–35^ The g.8168G>A mutation leads to an arginine to glutamine (R444Q) amino acid substitution that destabilizes the heme-binding domain.^36^ Unlike wild-type human *CYP1B1* mRNA, injecting g.3976G>C, g.4490G>A, or g.8168G>A mutant *CYP1B1* mRNA into one-cell-stage zebrafish embryos did not significantly affect eye size, neural crest development, or ocular fissure closure (Supplementary Table S5; Figs. 8D, 8D', 8E, 8E', 8F, 8F') compared with control embryos. Thus, these studies demonstrated the evolutionary conservation of *CYP1B1* gene function between humans and zebrafish.

## Discussion

In primary infantile-onset glaucoma, mutations in the *CYP1B1* gene lead to increased intraocular pressure and subsequent vision loss resulting from damage to the optic nerve, cornea, and sclera.^1–3^ However, the mechanism by which *CYP1B1* regulates eye development has not been defined, and the in vivo substrate for this cytochrome p450 enzyme during embryogenesis has not previously been identified. In the present study, we used a zebrafish model to improve our understanding of the pathophysiology of primary infantile-onset glaucoma.

*CYP1B1* mutations associated with primary infantile-onset glaucoma have primarily been detected in the coding region, comprising the second and third exons of this gene.^3,37^ The CYP1B1 protein comprises an N-terminal membrane-binding domain, a short “hinge” region, and a large C-terminal cytosolic domain. Disease-causing point mutations frequently affect highly conserved regions in the cytosolic portion, including the heme-binding region, substrate-binding region, and the substrate access channel. For example, the g.4490G>A (E229K) mutation, one of the most commonly identified point mutations associated with primary infantile-onset glaucoma, destabilizes ionic interactions between helices in the substrate-binding domain, resulting in decreased protein activity.^3,30^ In the present study, the overexpression of either the zebrafish *cyp1b1* or human *CYP1B1* yielded a similar phenotype: colobomatous defects and abnormal jaw and PA formation. However, the injection of human mRNA containing clinically significant point mutations had less effect on eye and craniofacial development. The effects of the injection of wild type were rescued after knocking down endogenous Cyp1b1, demonstrating functional conservation between human and zebrafish forms of this gene and indicating that zebrafish can be used as a model for studying the in vivo function of *CYP1B1* in eye development.

In zebrafish, Cyp1b1 plays a role in metabolizing polycyclic aromatic hydrocarbons, such as 2,3,7,8-tetrachlorodibenzodioxin. *Cyp1b1* transcription is directly regulated via exposure to exogenous toxins through the activation of the aryl hydrocarbon receptor.^24,38^ However, this regulatory pathway is not activated until 72 hpf, indicating that early *cyp1b1* expression in the developing eye is regulated through developmental pathways, and this enzyme has an endogenous substrate. In vitro studies have shown that Cyp1b1 catalyzes both enzymatic steps in the conversion of retinol into RA.^5^ This mechanism represents an alternative pathway from the well-characterized canonical pathway, which uses alcohol dehydrogenases to convert retinol into retinal and retinaldehyde dehydrogenases to convert retinal into RA. In mice, chickens, and zebrafish, the developing eye expresses retinaldehyde dehydrogenases in the dorsal and ventral retina. The local synthesis of RA is counteracted by degradation enzymes (Cyp26a1, Cyp26b1, and Cyp26c1) expressed in the cranial and caudal retina.^6–9^ The specific expression of synthesis and degradation enzymes generates RA gradients, where the highest concentrations of RA are localized along the dorsal–ventral axis of the developing eye. Retinoic acid is critical for both craniofacial and ocular neural crest development. We previously demonstrated that decreased or increased RA levels disrupt neural crest contributions to the cornea, iris, jaw, and PA in zebrafish.^17^ The present study demonstrated that *cyp1b1* expression was regulated by alterations in RA levels and the overexpression of *cyp1b1* increased RA levels throughout developing zebrafish embryos. Further, the increase in RA as a result of *cyp1b1* overexpression decreased the microphthalmia induced through RA deficiency (Raldh2 MO). Cyp1b1 knockdown had minimal effect on RA expression and eye development. However, decreased Cyp1b1 expression improved the effects of increased RA as a result of the overexpression of *raldh2*. Taken together, *cyp1b1* may regulate RA synthesis in vivo through a secondary pathway that can be either up- or downregulated when the primary aldehyde dehydrogenase pathway is genetically or pharmacologically altered; however, the primary endogenous role of *cyp1b1* in eye development seems to be independent of RA.

A key function of *cyp1b1* may be the regulation of ocular fissure closure. *Cyp1b1* expression in the developing eye was localized to the retina and retinal pigment epithelium within and immediately surrounding the ocular fissures. Although the inferior ocular fissure is well defined, the closure of the superior fissure prior to its ventral counterpart in zebrafish is less well understood (Stach T, et al. *IOVS* 2013;54:ARVO E-Abstract 3362). *Cypb1* expression was correlated with the patency of ocular fissures, as *cypb1* expression is highest between 24 and 36 hpf, after which expression becomes attenuated and is undetectable at 60 hpf. This finding suggests a role for *cyp1b1* in maintaining the patency of ocular fissures. Ocular fissure closure is a complex process that requires proper optic vesicle evagination from the forebrain, specific spatial patterning of the optic cup along the dorsal–ventral and proximal–distal axes, breakdown of the basement membrane enveloping the anterior and posterior edges, and tissue fusion.^27,28,39,40^ In zebrafish embryos, the ventral fissure closure is initiated in the middle of the optic cup and proceeds anteriorly and posteriorly between 36 and 72 hpf.^41^ In the present study, the premature breakdown of laminin in the basement membrane was observed in Cyp1b1 MO knockdown embryos, suggesting premature ocular fissure closure compared with control MO-injected and uninjected embryos. Time-lapse imaging further revealed that the premature closure of the fissure disrupted neural crest migration adjacent to and through the ocular fissure into the anterior segment. In zebrafish embryos, premature fissure closure did not seem to affect neural crest contributions to the cornea and iris at 96 hpf; thus, we propose that this mechanism may account for the specific disruption of the neural crest–derived trabecular meshwork in primary infantile-onset glaucoma. In humans, neural crest cells travel in three separate waves, with the first wave generating the components of the cornea, the second wave contributing to the iris, and the third wave forming the trabecular meshwork and angle structures. While neural crest cells destined for the cornea and iris enter the anterior segment, the early closure of the ocular fissure may prevent neural crest specifically destined for the trabecular meshwork from migrating into the anterior segment. Differences in the effects on the neural crest in *cyp1b1* mutant zebrafish, mice, and chickens may reflect species differences in the number of waves of neural crest migration (e.g., two in chickens and one in mice) and anatomic differences in aqueous outflow pathways.^42,43^ Taken together, the regulation of ocular fissure patency may explain the mechanism by which *cyp1b1* affects neural crest contributions to the anterior segment of the eye.

Interestingly, overexpression of *cyp1b1* resulted in large colobomas. *CYP1B1* mutations have not been reported in cases of inferior colobomas, but this gene is not included in the typical genetic screens used in clinical testing. Notably, a *CYP1B1* mutation was identified in a patient with a rare superior coloboma (Stach T, et al. *IOVS* 2013;54:ARVO E-Abstract 3362). However, whether this variant is a gain-of-function mutation remains unknown, indicating that additional studies are required to further identify and assess mutations in human *CYP1B1* in cases of colobomas.

Given the timing of the changes in the expression of *shh*, *pax6*, and *vsx2* and the increased apoptosis in the inferior ocular fissure, we hypothesized that *cyp1b1* overexpression had an early effect on the spatial patterning of the optic cup. In humans, heterozygous mutations in *SHH* are associated with holoprosencephaly and a spectrum of eye malformations, including anophthalmia, microphthalmia, and colobomas.^44–46^ *Shh* is expressed in the floor plate of the diencephalon and is required for patterning the optic cup through the specific spatial expression of transcription factors.^47^ *Shh* regulates *Vax2* and *Pax2* in the optic stalk and ventral neural retina to maintain the expression of these transcription factors within the more proximal structures of the eye.^48,49^ Mice with *Vax2* or *Pax2* mutations show the inhibition of basement membrane breakdown in the fissure, resulting in colobomas. In contrast, *Shh* inhibits *Pax6*, restricting its expression to the distal optic cup, which is critical for lens induction from the surface ectoderm.^50,51^ Human mutations in *PAX6* are most commonly associated with aniridia (OMIM 706108), an autosomal dominant congenital eye disease characterized by cataract, limbal stem cell deficiency, glaucoma, and hypoplasia of the iris, optic nerve, and fovea. While large disruptions in gene function resulting from frameshift or nonsense mutations lead to these widespread ocular defects,^52,53^ rare missense *PAX6* mutations have been associated with colobomas.^54^ In mice, *Pax6* regulates other genes associated with colobomas, such as *Vsx2* (*Chx10*), *Maf1*, and *Six3*.^55–57^ The current studies showed that in zebrafish, *cyp1b1* overexpression increased *shha* gene expression in the midline floor plate, while decreasing *pax6a*, *pax6b*, and *vsx2* gene expression in the developing retina. Notably, RA is also a key regulator of ocular fissure closure, as both increased and decreased RA result in colobomas in mice and zebrafish.^58–61^ Exposure to exogenous RA inhibits *Shh* expression in the midline floor plate.^62^ We observed that the overexpression of *cyp1b1* not only increased RA levels throughout the developing head and eye, but also increased *shha* expression. Furthermore, genetically or pharmacologically decreasing endogenous RA synthesis to compensate for *cyp1b1* overexpression did not rescue the observed colobomatous defects. It is unclear whether these signaling pathways are affected only in the overexpression state or represent normal endogenous targets of *cyp1b1*. We did not observe alterations in the levels of *shh*, *pax6*, or *vsx2* with decreased Cyp1b1, suggesting that there are additional targets of Cyp1b1 that regulate fissure patency. Taken together, the results of the present study provide evidence that the overexpression of *cyp1b1* regulates ocular fissure closure through an RA-independent mechanism involving *shh* and *pax6*.

In summary, we investigated the mechanisms by which *Cyp1b1* regulates eye development. The functions of the zebrafish and human forms of the *CYP1B1* gene were evolutionarily conserved. *Cyp1b1* plays a primary role in regulating ocular fissure closure, which indirectly regulates neural crest migration, and this mechanism was independent of RA. These results improve the current understanding of the complex signals that regulate eye development and, importantly, provide insight into the molecular pathogenesis of primary infantile-onset glaucoma.

## Supplementary Material

Supplement 1

Supplement 2

## References

[1] Aponte EP, Diehl N, Mohney BG. Inicidence and clinical characteristics of childhood glaucoma: a population-based study. *Arch Ophthalmol*. 2010; 128: 478–482. 20385945 10.1001/archophthalmol.2010.41 PMC2885872

[2] Papadopoulos M, Cable N, Rahi J, Khaw PT; BIG Eye Study Investigators. The British Infantile and Childhood Glaucoma (BIG) Eye Study. *Invest Ophthalmol Vis Sci*. 2007; 48: 100–106. 10.1167/iovs.06-1350 17724193

[3] Li N, Zhou Y, Du L, Wei M, Chen X. Overview of cytochrome p450 1B1 gene mutations in patients with primary congenital glaucoma. *Exp Eye Res*. 2011; 93: 572–579. 21854771 10.1016/j.exer.2011.07.009

[4] Libby RT, Smiths RS, Savinova OV, Modification of ocular defects in mouse developmental glaucoma models by tyrosinase. *Science*. 2003; 299: 1578–1581. 12624268 10.1126/science.1080095

[5] Chambers D, Wilson L, Maden M, Lumsden A. RALDH-independent generation of retinoic acid during vertebrate embryogenesis by CYP1B1. *Development*. 2007; 134: 1369–1383. 17329364 10.1242/dev.02815

[6] McCaffery P, Wagner E, O'Neil J, Petkovich M, Drager UC. Dorsal and ventral retinal territories defined by retinoic acid synthesis, break-down and nuclear receptor expression. *Mech Dev*. 1999; 82: 119–130. 10354476 10.1016/s0925-4773(99)00022-2

[7] Grandel H, Brand M. Zebrafish limb development is triggered by a retinoic acid signal during gastrulation. *Dev Dyn*. 2011; 240: 1116–1126. 21509893 10.1002/dvdy.22461

[8] Cvekl A, Wang W-L. Retinoic acid signaling in mammalian eye development. *Exp Eye Res*. 2009; 89: 280–291. 19427305 10.1016/j.exer.2009.04.012 PMC2756743

[9] Aulehla A, Pourquie O. Signaling gradients during paraxial mesoderm development. *Cold Spring Harb Perspect Biol*. 2010; 2: a000869. 20182616 10.1101/cshperspect.a000869 PMC2828275

[10] Bohnsack BL, Kasprick D, Kish PE, Goldman D, Kahana A. A zebrafish model of Axenfeld-Rieger Syndrome reveals that *pitx2* regulation by retinoic acid is essential for ocular and craniofacial development. *Invest Ophthalmol Vis Sci*. 2012; 53: 7–22. 22125274 10.1167/iovs.11-8494 PMC3292384

[11] Bohnsack BL, Kahana A. Thyroid hormone and retinoic acid interact to regulate zebrafish craniofacial neural crest development. *Dev Biol*. 2013; 373: 300–309. 23165295 10.1016/j.ydbio.2012.11.005 PMC3534885

[12] Trainor PA. Specification of neural crest cell formation and migration in mouse embryos. *Semin Cell Dev Biol*. 2005; 16: 683–693. 16043371 10.1016/j.semcdb.2005.06.007

[13] Gage PJ, Rhoades W, Prucka SK, Hjalt T. Fate maps of neural crest and mesoderm in the mammalian eye. *Invest Ophthalmol Vis Sci*. 2005; 46: 4200–4208. 16249499 10.1167/iovs.05-0691

[14] Strungaru MH, Dinu I, Walter MA. Genotype-phenotype correlations in Axenfeld-Rieger malformation and glaucoma patients with FOXC1 and PITX2 mutations. *Invest Ophthalmol Vis Sci*. 2007; 48: 228–237. 17197537 10.1167/iovs.06-0472

[15] Harissi-Dagher M, Colby K. Anterior segment dysgenesis: Peters anomaly and sclerocornea. *Int Ophthalmol Clin*. 2008; 48: 35–42. 18427259 10.1097/IIO.0b013e318169526c

[16] Vincent A, Billingsley G, Priston M, Phenotypic heterogeneity of CYP1B1: mutations in a patient with Peters Anomaly. *J Med Genet*. 2001; 36: 152–155. 10.1136/jmg.38.5.324 PMC1734880 11403040

[17] Bohnsack BL, Gallina D, Thompson H, Development of extraocular muscles require early signals from periocular neural crest and the developing eye. *Arch Ophthalmol*. 2011; 129: 1030–1041. 21482859 10.1001/archophthalmol.2011.75 PMC3248700

[18] Bohnsack BL, Kahana A. Phenothiourea sensitizes zebrafish cranial neural crest and extraocular muscle developmnt to changes in retinoic acid and insulin-like growth factor signaling. *PLoS One*. 2011; 6: e22991. 21886774 10.1371/journal.pone.0022991 PMC3158757

[19] Kimmel CB, Ballard WW, Kimmel SR, Ullmann BB, Schilling TF. Stages of embryonic development of the zebrafish. *Dev Dyn*. 1995; 203: 253–310. 8589427 10.1002/aja.1002030302

[20] Curran K, Raible DW, Lister JA. Foxd3 controls melanophore specification in the zebrafish neual crest by regulation of Mitf. *Dev Biol*. 2009; 332: 408–417. 19527705 10.1016/j.ydbio.2009.06.010 PMC2716409

[21] Dutton K, Dutton JR, Pauliny A, Kelsh RN. A morpholino phenocopy of the colourless mutant. *Genesis*. 2001; 30: 188–189. 11477705 10.1002/gene.1062

[22] Dutton KA, Pauliny A, Lopes SS, Zebrafish colourless encodes sox10 and specifies non-ectomesenchymal neural crest fates. *Development*. 2001; 128: 4113–4125. 11684650 10.1242/dev.128.21.4113

[23] Chawla B, Schley E, Williams AL, Bohnsack BL. Retinoic acid and *pitx2* regulate early neural crest survival and migration in craniofacial and ocular development. *Birth Defects Res B Dev Reprod Toxicol*. 2016; 107: 126–135. 27175943 10.1002/bdrb.21177

[24] Timme-Laragy AR, Noyes PD, Buhler DR, Di Giulio RT. CYP1B1 knockdown does not alter synergistic developmental toxicity of polycyclic aromatic hydrocarbons in zebrafish (*Danio rerio*). *Mar Environ Res*. 2008; 66: 85–87. 18378296 10.1016/j.marenvres.2008.02.030 PMC2516962

[25] Barthel LK, Raymond PA. In situ hybridization studies of retinal neurons. *Methods Enzymol*. 2000; 316: 579–590. 10800703 10.1016/s0076-6879(00)16751-5

[26] Williams AL, Bohnsack BL. Neural crest derivatives in ocular development: discerning the eye of the storm. *Birth Defects Res C Embryo Today*. 2015; 105: 87–95. 26043871 10.1002/bdrc.21095 PMC5262495

[27] Gregory-Evans CY, Williams MJ, Halford S, Gregory-Evans K. Ocular coloboma: a reassessment in the age of molecular neuroscience. *J Med Genet*. 2004; 41: 881–891. 15591273 10.1136/jmg.2004.025494 PMC1735648

[28] Gongal PA, French CR, Waskiewicz AJ. Aberrant forebrain signaling during early development underlies the generation of holoprosencephaly and coloboma. *Biochim Biophys Acta*. 2011; 1912: 390–401. 10.1016/j.bbadis.2010.09.005 20850526

[29] Bohnsack BL, Gallina D, Kahana A. Phenothiourea sensitizes zebrafish cranial neural crest and extraocular muscle developmnt to changes in retinoic acid and insulin-like growth factor signaling. *PLoS One*. 2011; 6: e22991. 21886774 10.1371/journal.pone.0022991 PMC3158757

[30] Achary MS, Reddy AB, Chakrabarti S, Disease-causing mutations in proteins: structural analysis of the CYP1B1 mutations causing primary congenital glaucoma in humans. *Biophys J*. 2006; 91: 4329–4339. 16963504 10.1529/biophysj.106.085498 PMC1779944

[31] Stoilov I, Akarsu AN, Alozie I, Sequence analysis and homology modeling suggest that primary congenital glaucoma on 2p21 results from mutations disrupting either the hinge region or the conserved core structures of cytochrome P450 1B1. *Am J Hum Genet*. 1998; 62: 573–584. 9497261 10.1086/301764 PMC1376958

[32] Acharya M, Huang L, Fleisch VC, Allison WT, Walter MA. A complex regulatory network of transcription factors critical for ocular development and disease. *Hum Mol Genet*. 2011; 20: 1610–1624. 21282189 10.1093/hmg/ddr038

[33] Badeeb OM, Micheal S, Koenekoop RK, den Hollander AI, Hedrawi MT. CYP1B1 mutations in patients with primary congenital glaucoma from Saudia Arabia. *BMC Med Genet*. 2014; 15: 109. 25261878 10.1186/s12881-014-0109-2 PMC4258803

[34] Chavarria-Soley G, Sticht H, Akillu E, Mutations in CYP1B1 cause primary congenital glaucoma by reduction of either activity or abundance of the enzyme. *Hum Mutat*. 2008; 29: 1147–1153. 18470941 10.1002/humu.20786

[35] Hollander DA, Sarfarazi M, Stoilov I, Genotype and phenotype correlations in congenital glaucoma: CYP1B1 mutations, goniodysgenesis, and clinical characteristics. *Am J Ophthalmol*. 2006; 142: 993–1004. 17157584 10.1016/j.ajo.2006.07.054

[36] Mashima Y, Suzuki Y, Sergeev Y, Novel cytochrome P4501B1 (CYP1B1) gene mutations in Japanese patients with primary congenital glaucoma. *Invest Ophthalmol Vis Sci*. 2001; 42: 2211–2216. 11527932

[37] Lloret-Vilaspasa F, Jensen HJ, de Roos K, Retinoid signalling is required for information transfer from mesoderm to neuroectoderm during gastrulation. *Int J Dev Biol*. 2010; 54: 599–608. 20209433 10.1387/ijdb.082705fl

[38] Yin H-C, Tseng H-P, Chung H-Y, Influence of TCDD on zebrafish CYP1B1 transcription during development. *Toxicol Sci*. 2008; 103: 158–168. 18308702 10.1093/toxsci/kfn035

[39] Matsushima D, Heavner W, Pevny LH. Combinatorial regulation of optic cup progenitor cell fate by SOX2 and PAX6. *Development*. 2011; 138: 443–454. 21205789 10.1242/dev.055178 PMC3014633

[40] Reza HM, Takahashi Y, Yasuda K. Stage-dependent expression of Pax6 in optic vesicle/cup regulates patterning genes through signaling molecules. *Differentiation*. 2007; 75: 726–736. 17381541 10.1111/j.1432-0436.2007.00168.x

[41] Pillai-Kastoori L, Wen W, Wilson SG, Sox11 is required to maintain proper levels of hedgehog signaling during vertebrate ocular morphogenesis. *PLoS Genet*. 2014; 10: e1004491. 25010521 10.1371/journal.pgen.1004491 PMC4091786

[42] Hay ED, Revel JP. Fine structure of the developing avian cornea. *Monogr Dev Biol*. 1969; 1: 1–144. 5407672

[43] Pei YF, Rhodin JA. The prenatal development of the mouse eye. *Anat Rec*. 1970; 168: 105–125. 5469558 10.1002/ar.1091680109

[44] Schimmenti LA, de la Cruz J, Lewis RA, Novel mutation in sonic hedgehog in non-syndrome colobomatous microphthalmia. *Am J Med Genet*. 2003; 116A: 215–221. 12503095 10.1002/ajmg.a.10884

[45] Geng X, Speirs C, Lagutin O, Haploinsufficiency of Six3 fails to activate Sonic hedgehog expression in the ventral forebrain and causes holoprosencephaly. *Dev Cell*. 2008; 15: 236–247. 18694563 10.1016/j.devcel.2008.07.003 PMC2597207

[46] Nanni L, Ming JE, Bocian M, The mutational spectrum of the sonic hedgehog gene in holoprosencephaly: SHH mutations cause a significant proportion of autosomal dominant holoprosencephaly. *Hum Mol Genet*. 1999; 8: 2479–2488. 10556296 10.1093/hmg/8.13.2479

[47] Ingham PW, Placzek M. Orchestrating ontogenesis: variations on a theme by sonic hedgehog. *Nat Rev Genet*. 2006; 7: 841–850. 17047684 10.1038/nrg1969

[48] Barbieri AM, Broccoli V, Bovolenta P, Vax2 inactivation in mouse determines alteration of the eye dosal-ventral axis, misrouting of the optic fibres and eye coloboma. *Development*. 2002; 129: 805–813. 11830579 10.1242/dev.129.3.805

[49] Torres M, Gomez-Pardo E, Gruss P. Pax2 contributes to inner ear patterning and optic nerve trajectory. *Development*. 1996; 122: 3381–3391. 8951055 10.1242/dev.122.11.3381

[50] Macdonald R, Barth KA, Xu Q, Holder N, Mikkola I, Wilson SW. Midline signalling is required for Pax gene regulation and patterning of the eyes. *Development*. 1995; 121: 3267–3278. 7588061 10.1242/dev.121.10.3267

[51] Canto-Soler MV, Adler R. Optic cup and lens development requires Pax6 expression in the early optic vesicle during a narrow time window. *Dev Biol*. 2006; 294: 119–132. 16564518 10.1016/j.ydbio.2006.02.033

[52] Hingorani M, Hanson I, van Heyningen V. Aniridia. *Eur J Hum Genet*. 2012; 20: 1011–1017. 22692063 10.1038/ejhg.2012.100 PMC3449076

[53] Lee H, Khan R, O'Keefe M. Aniridia: current pathology and management. *Acta Ophthalmol*. 2008; 86: 708–715. 18937825 10.1111/j.1755-3768.2008.01427.x

[54] Azuma N, Yamaguchi Y, Handa H, Mutations of the PAX6 gene detected in patients with a variety of optic-nerve malformations. *Am J Hum Genet*. 2003; 72: 1565–1570. 12721955 10.1086/375555 PMC1180317

[55] Ferda-Percin E, Ploder LA, Yu JJ, Human microphthalmia associated with mutations in the retinal homeobox gene CHX10. *Nat Genet*. 2000; 25: 397–401. 10932181 10.1038/78071

[56] Jamieson RV, Perveen R, Kerr B, Domain disruption and mutation of the bZIP transcription factor, MAF, associated with cataract, ocular anterior segment dysgenesis and coloboma. *Hum Mol Genet*. 2002; 11: 33–42. 11772997 10.1093/hmg/11.1.33

[57] Wallis EC, Rosessler E, Hehr U, Mutations in the homeodomain of the human SIX3 gene cause holoprosencephaly. *Nat Genet*. 1999; 22: 196–198. 10369266 10.1038/9718

[58] Stull DL, Wikler KC. Retinoid-dependent gene expression regulates early morphological events in the development of the murine retina. *J Comp Neurol*. 2000; 417: 289–298. 10683604 10.1002/(sici)1096-9861(20000214)417:3<289::aid-cne3>3.0.co;2-s

[59] Marsh-Armstrong N, McCaffery P, Gilbert W, Dowling JE, Drager UC. Retinoic acid is necessary for development of the ventral retina in zebrafish. *Proc Natl Acad Sci U S A*. 1994; 91: 7286–7290. 8041782 10.1073/pnas.91.15.7286 PMC44384

[60] Molotkov A, Molotkova N, Duester G. Retinoic acid guides eye morphogenetic movements via paracrine signaling but is unnecessary for retinal dorsoventral patterning. *Development*. 2006; 133: 1901–1910. 16611695 10.1242/dev.02328 PMC2833011

[61] See AW, Clagett-Dame M. The temporal requirement for vitamin A in the developing eye: mechanism of action in optic fissure closure and new roles for the vitamin in regulating cell proliferation and adhesion in the embryonic retina. *Dev Biol*. 2009; 325: 94–105. 18955041 10.1016/j.ydbio.2008.09.030

[62] Helms JA, Kim CH, Hu D, Minkoff R, Thaller C, Eichele G. Sonic hedgehog participates in craniofacial morphogenesis and is down-regulated by teratogenic doses of retinoic acid. *Dev Biol*. 1997; 187: 25–35. 9224671 10.1006/dbio.1997.8589

