# Supplementary material for: Cyp1b1 Regulates Ocular Fissure Closure Through a Retinoic Acid–Independent Pathway
Source: Invest Ophthalmol Vis Sci. 2017 Feb;58(2):1084–97. doi: 10.1167/iovs.16-20235 (PMC5308778; doi:10.1167/iovs.16-20235)
Supplement: Supplement 1 [file iovs-58-02-29_s01.pdf]

Supplemental Table 1

Target	Morpholino	Control	Conc./Amount Injected	Verification Methods and Ref
Cyp1b1 (ATG)	TCTCAGAGCCAGCAGGACATCCATC	TCTGACAGCGAGCAGCAGATCCATC (5 bp mismatch)	2.1 ng/nl<br>2.1-4.2 ng	Protein confirmation*,[25]<br>Complementary mRNA injection (current studies)
Cyp1b1 (5' UTR)	ATTTGCACGTCTCGGAAATCAATGC	ATATCCACCTCTCGCAAATCAATCC (5 bp mismatch)	2.3 ng/nl<br>2.3-4.6 ng	Confirmation of MO phenotype (current studies)
Raldh2 (ATG)	GTTCAACTTCACTGGAGGTCATC	CCTCTTACCTCAGTTACAATTTATA (standard Gene-Tools control MO)	2.1 ng/nl<br>2.1-4.2 ng	Protein confirmation (current studies)
Raldh2 (5' UTR)	CTGCCGATGTGCGATTACAACTTTG	CTCCCCATCTGCCATTAGAACTTTG (5 bp mismatch)	2.3 ng/nl<br>2.3-4.6 ng	Protein confirmation (current studies)<br>Complementary mRNA injection (current studies)<br>Confirmation of MO phenotype (current studies)

*The anti-Cyp1b1 antibody used in Timme-Laragy et al.[25] (confirming decreased protein expression with MO) is not available. The commercially available antibodies against human CYP1B1, for cross-reactivity with zebrafish was predicted (Abcam Ab78044 and Ab157578), did not detect the zebrafish form by Western blotting (Fig. 7F).

Supplemental Table 2

Human *CYP1B1* missense mutations

**DNA Mutation GenBank U56438**	**Protein**	**Protein Structure**	**References**
3976G>C, Exon 2	W57C	Hinge Region	3, 35
4490G>A, Exon 2	E229K	Cytosolic; Substrate-Binding Region	3, 34, 37-39
8168G>A, Exon 3	R444Q	Cytosolic; Heme-Binding Region	3, 40

Supplemental Table 3

Group	Craniofacial Abnormalities/ Developmental Delay	Injected Embryos	p value
Uninjected	8.3 ±6.3%	1479	
Cyp1b1 ATG Mismatch MO	12.1 ± 9.8%	277	0.98 (vs. Uninjected)
Cyp1b1 ATG MO	20.5 ± 12.1%	299	0.56 (vs. Cyp1b1 ATG Mismatch MO)
Cyp1b1 5' UTR Mismatch MO	21.0 ± 7.1%	692	0.10 (vs. Uninjected)
Cyp1b1 5' UTR MO	26.8 ± 11.1%	845	0.97 (vs. Cyp1b1 5' UTR Mismatch MO)
GFP mRNA	5.7 ± 4.9%	326	0.99 (vs. Uninjected)
z *cyp1b1* mRNA	43.8 ± 12.1% (Delay)<br><br>38.7 ± 8.5% (Coloboma)	814	<0.0001 (vs. *GFP* mRNA)<br><br>0.0001 (vs. *GFP* mRNA)
Cyp1b1 ATG MO + z *cyp1b1* mRNA	20.9 ± 6.0%	179	0.002 (vs. z *cyp1b1* RNA)<br>0.99 (vs. Cyp1b1 ATG MO)
Cyp1b1 5'UTR MO + z *cyp1b1* mRNA	27.9 ± 4.8%	478	0.03 (vs. z *cyp1b1* RNA)<br>0.86 (vs. Cyp1b1 UTR MO)
raldh2 mRNA	56.2 ± 17.7%	182	0.002 (vs. *GFP* mRNA)
Raldh2 MO	80.4 ± 5.9%	168	0.0001 (vs. Uninjected)
Raldh2 MO + *raldh2* mRNA	20.5 ± 6.8%	181	0.01 (vs. *raldh2* mRNA)<br>0.0004(vs. Raldh2 MO)
Cyp1b1 ATG MO + *raldh2* RNA	31.4 ± 9.0%	169	0.04 (vs. *raldh2* mRNA)<br>0.35 (vs. Cyp1b1 ATG MO)
cyp1b1 mRNA + Raldh2 ATG MO	53.5 ± 7.7% (Delay)<br><br>30.0 ± 8.7% (Coloboma)	171	0.0001 (vs. Raldh2 MO)<br>0.49 (vs. z *cyp1b1* mRNA)<br><br>0.17 (vs. z *cyp1b1* mRNA)
Uninjected + 0.003% PTU	17.6 ± 7.0%	293	0.99 (vs. Uninjected)
Cyp1b1 ATG Mismatch MO +	26.3 ± 3.5%	309	0.28 (vs. Uninjected + 0.3% PTU)

0.003% PTU			
Cyp1b1 ATG MO + 0.003% PTU	29.0 ± 7.5%	322	0.85 (vs. Cyp1b1 Mismatch MO + 0.3% PTU)
Uninjected in *casper*	8.4 ± 10.4%	243	0.99 (vs. Uninjected)
Cyp1b1 ATG Mismatch MO in *casper*	19.4 ± 12.8%	240	0.74 (vs. Uninjected in *casper*)
Cyp1b1 ATG MO in *casper*	25.0 ± 11.0%	250	0.71 (vs. Cyp1b1 ATG Mismatch MO in *casper*)

Supplemental Table 4

**Group**	**Ocular Fissure Defect/Coloboma**	**Treated/ Injected Embryos**	**p value**
GFP RNA			
Untreated	3.3 ± 1.0%	150	
1μM DEAB	28.0 ± 7.0%	161	0.10 (vs. GFP-injected Untreated)
2μM DEAB	38.0 ± 9.4%	160	0.008 (vs. GFP-injected Untreated)
5μM DEAB	55.2 ± 4.6%	153	<0.0001 (vs. GFP-injected Untreated)
cyp1b1 RNA			
Untreated	45.2 ± 9.8%	130	
1μM DEAB	82.2 ± 13.4%	121	0.0007 (vs. *cyp1b1* mRNA injected Untreated)<br><0.0001 (vs. GFP-injected 1μM DEAB treated)
2μM DEAB	84.5 ± 14.4%	130	0.0003 (vs. *cyp1b1* mRNA injected Untreated)<br>0.0002 (vs. GFP-injected 2μM DEAB treated)
5μM DEAB	100%	109	<0.0001 (vs. *cyp1b1* mRNA injected Untreated)<br>0.0004 (vs. GFP-injected 5μM DEAB treated)

Supplemental Table 5

**Group**	**Ocular Fissure Defect/ Coloboma**	**Injected Embryos**	**p value**
Uninjected	7.0 ± 5.3%	322	
Human *CYP1B1* mRNA	71.8 ± 17.9%	493	0.0003 (vs. uninjected)
Human *CYP1B1* mRNA + Cyp1b1 MO	36.4 ± 17.0%	271	0.06(vs. Human *CYP1B1* mRNA)
Human 3976G>C *CYP1B1* mRNA	16.1 ± 4.7%	320	0.0008(vs. Human *CYP1B1* mRNA)
Human 4490G>A *CYP1B1* mRNA	16.1 ± 7.4%	331	0.0032(vs. Human *CYP1B1* mRNA)
Human 8168G>A *CYP1B1* mRNA	22.7 ± 9.7%	306	0.0008(vs. Human *CYP1B1* mRNA)
