# Supplementary material for: Cyp1b1 Regulates Ocular Fissure Closure Through a Retinoic Acid–Independent Pathway
Source: Invest Ophthalmol Vis Sci. 2017 Feb;58(2):1084–97. doi: 10.1167/iovs.16-20235 (PMC5308778; doi:10.1167/iovs.16-20235)
Supplement: Supplement 2 [file iovs-58-02-29_s02.pdf]

Supplemental Fig 1

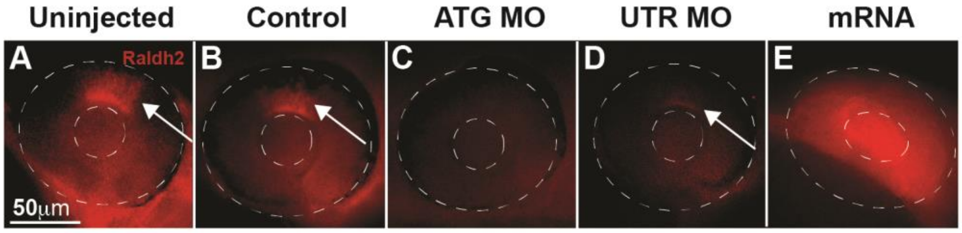

Supplemental Figure 1. Raldh2 MO knockdown decreased protein

Wholemount immunostaining for Raldh2 showed expression in the dorsal retina in 24 hpf uninjected (A) and control-injected (B) embryos. Injection of Raldh2 ATG (C) or 5’ UTR (D) MO decreased Raldh2 protein expression in the eye. Injection of mRNA (E) increased *raldh2* expression in the eye.

Supplemental Fig 2

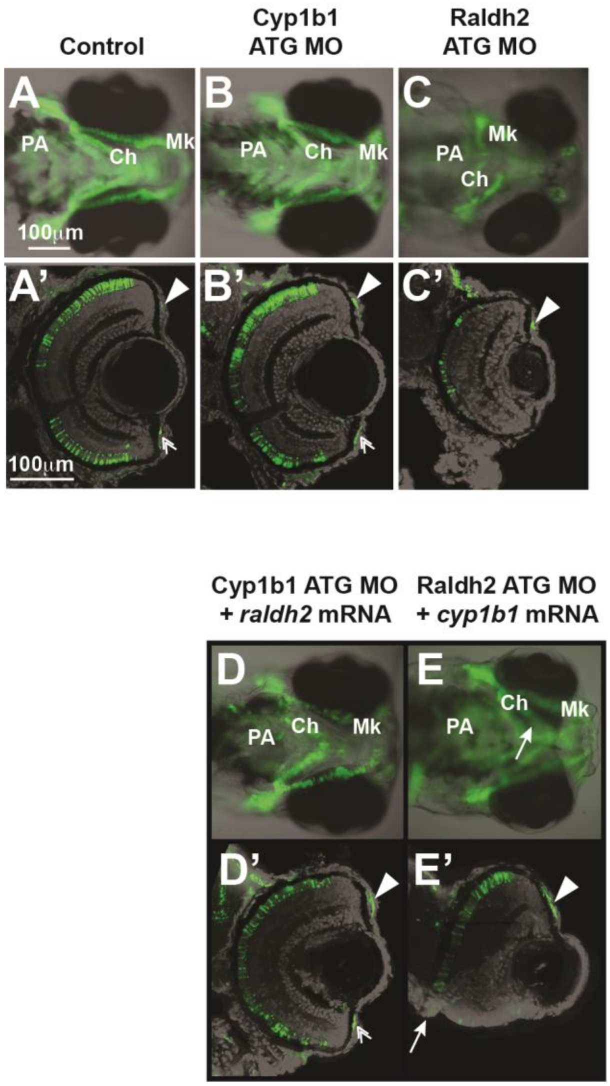

Supplemental Figure 2. Overexpression of *cyp1b1* inhibited ocular fissure closure via an RA-independent pathway.

Live images of 96 hpf Tg(*sox10:EGFP*) embryos and sections of 96 hpf Tg(*foxd3:GFP*) showed that a second Cyp1b1 ATG targeted MO had minimal effect on cranial neural crest development (B) and overall eye development (B') compared to controls (A, A'). A second Raldh2 ATG targeted MO inhibited pharyngeal arch development, disrupted ceratohyal and Meckel's cartilage formation (C) and decreased eye size

(C'). Cyp1b1 ATG MO eye size (D') and neural crest-derived pharyngeal arch, ceratohyal, and Meckel's cartilage formation (D) in embryos injected with *raldh2* mRNA. Raldh2 ATG MO knockdown improved neural crest-derived pharyngeal arch, ceratohyal, and Meckel's cartilage formation (E), but did not rescue inferior ocular fissure closure (arrows, E') in embryos injected with *cyp1b1* mRNA.

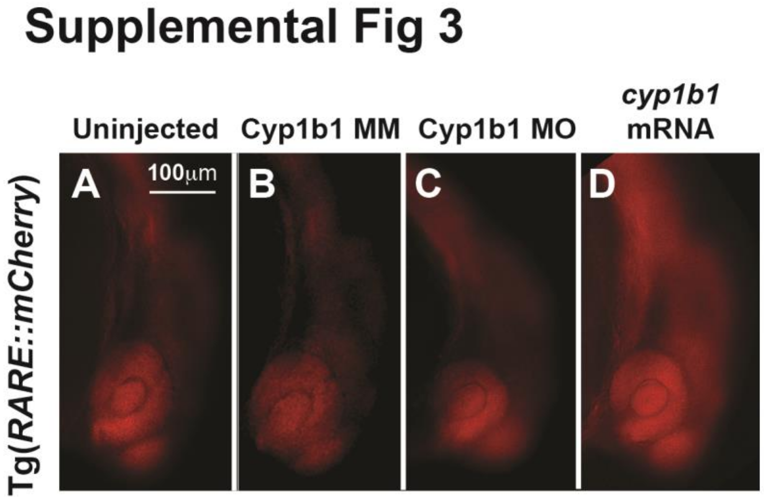

Supplemental Figure 3. Cyp1b1 regulated RA activity.

Live imaging of the Tg(*RARE:mCherry*) reporter line in the red channel alone showed areas of high RA activity in the eye and prosencephalon in uninjected (A) and control-injected (B) embryos. MO knockdown of Cyp1b1 (C) did not show gross changes in RA activity, while the overexpression of *cyp1b1* through mRNA injection diffusely increased mCherry expression at 24 hpf throughout the craniofacial region (D).

Supplemental Fig 4

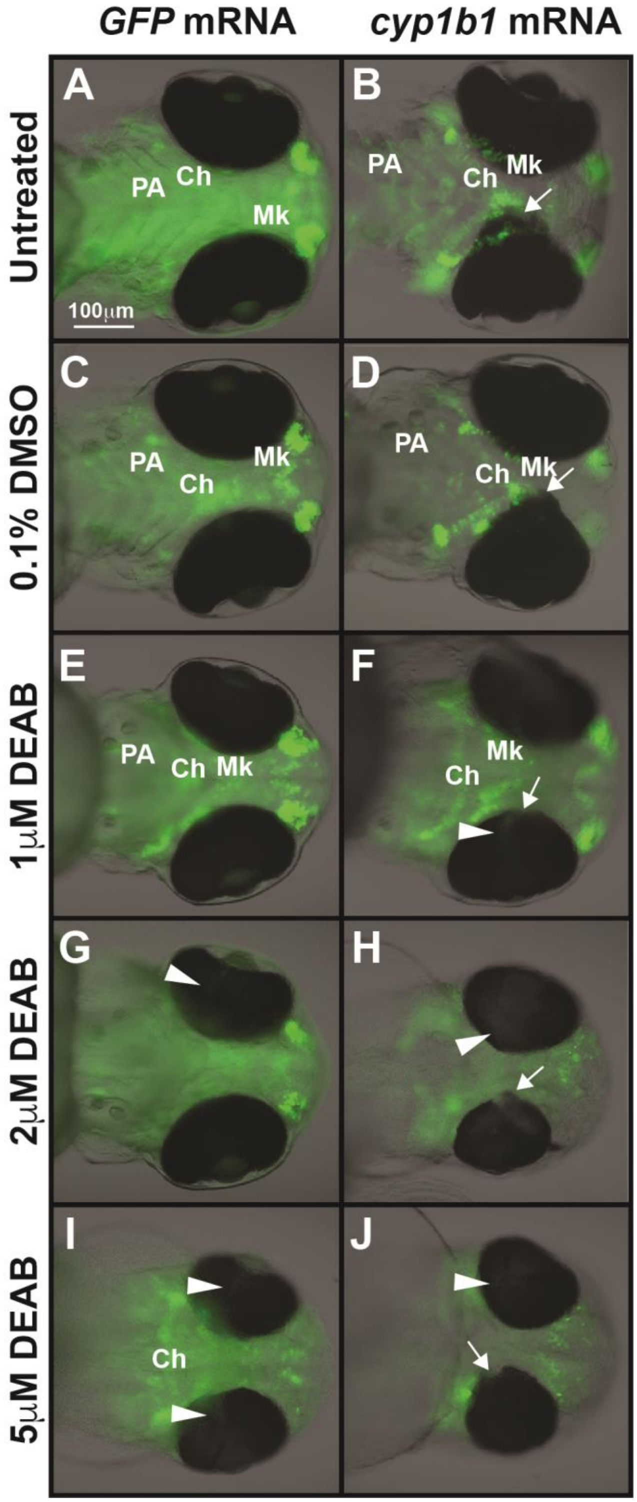

Supplemental Figure 4. Progressive inhibition of RA synthesis did not rescue defects associated with *cyp1b1* overexpression.

Live images of Tg(*sox10:EGFP*) embryos injected with *EGFP* mRNA showed that increasing concentrations of DEAB [1 μM (E), 2 μM (G), or 5 μM (I)] progressively inhibited pharyngeal arch ("PA"), ceratohyal ("Ch"), and Meckel's ("Mk") cartilage formation compared with DMSO control (C) and uninjected (A) embryos. In addition, higher concentrations of DEAB (5 μM) delayed ocular fissure closure (arrowheads), resulting in smaller eyes. Treatment of *cyp1b1* mRNA-injected embryos with increasing concentrations of DEAB [1 μM (E), 2 μM (G), or 5 μM (I)] did not rescue ocular fissure (arrowheads) and eye wall (arrows) defects compared with DMSO control-treated (D) or untreated (B) embryos.
